# A revision of *Prolimulus woodwardi* Fritsch, 1899 with comparison to other highly paedomorphic belinurids

**DOI:** 10.7717/peerj.10980

**Published:** 2021-03-08

**Authors:** Lorenzo Lustri, Lukáš Laibl, Russell D.C. Bicknell

**Affiliations:** 1 Institute of Earth Sciences, University of Lausanne, Geopolis, Lausanne, Switzerland; 2 Institute of Geology and Palaeontology, Faculty of Science, Charles University, Prague, Czech Republic; 3 Institute of Geology of the Czech Academy of Sciences, Prague, Czech Republic; 4 Palaeoscience Research Centre, School of Environmental and Rural Science, University of New England, Armidale, New South Wales, Australia

**Keywords:** Xiphosurida, Belinuridae, Carboniferous, Heterochrony, Epibiota

## Abstract

Xiphosurida is an ingroup of marine Euchelicerata often referred to as “living fossils”. However, this oxymoronic term is inapplicable for Paleozoic and early Mesozoic forms, as during these periods the group experienced notable evolutionary radiations; particularly the diverse late Palaeozoic clade Belinurina. Despite the iconic nature of the group, select species in this clade have been left undescribed in the light of recent geometric morphometric and phylogenetic considerations and methodologies. To this end, we re-describe *Prolimulus woodwardi* Fritsch, 1899 using new and type specimens to reveal more details on appendage anatomy and possible ecology. Furthermore, we present geometric morphometric and phylogenetic analyses that uncover relationships between *P. woodwardi* and other belinurids without genal spines. Both approaches highlight that a clade containing *Prolimulus* Fritsch, 1899, *Liomesaspis* Raymond, 1944, *Alanops* Racheboeuf, Vannier & Anderson, 2002 and *Stilpnocephalus* Selden, Simonetto & Marsiglio, 2019 may exist. While we do not erect a new group to contain these genera, we note that these genera exemplify the extreme limits of the Belinurina radiation and a peak in horseshoe crab diversity and disparity. This evidence also illustrates how changes in heterochronic timing are a key evolutionary phenomenon that can drive radiations among animals.

## Introduction

Xiphosurida, are an extant group of euchelicerates with an extensive fossil record spanning most of the Phanerozoic (Van Roy, Briggs & Gaines, 2015). They are often referred to as “living fossils” (Størmer, 1952), considered examples of stabilomorphism (Kin & Błażejowski, 2014) and morphological conservatism (Bicknell & Pates, 2020). Such statements are mostly applicable to the late Mesozoic and Cenozoic forms (Avise, Nelson & Sugita, 1994; Rudkin & Young, 2009; Kin & Błażejowski, 2014; Lamsdell & McKenzie, 2015; Błazejowski, Gieszcz & Tyborowski, 2016; Bicknell & Pates, 2019; Bicknell et al., 2019b; Bicknell & Pates, 2019). Conversely, most Paleozoic and early Mesozoic forms record evolutionary exploration (Bicknell, 2019; Bicknell, Amati & Hernández, 2019; Bicknell et al., 2019a; Bicknell, Naugolnykh & Brougham, 2020; Bicknell et al., in press; Bicknell, Hecker & Heyng , 2021; Bicknell & Pates, 2020). The evolutionary history of these earlier species illustrate morphological plasticity and exploration of different ecological niches (Lamsdell, 2016; Lamsdell, 2020a; Bicknell et al., 2019b). Belinurina—a clade containing Belinuridae—is a particularly diverse group known from the Carboniferous and Permian that successfully colonized freshwater environments. Belinurids have been considered at length (Størmer, 1952; Eldredge, 1974; Anderson & Selden, 1997; Haug et al., 2012; Haug & Haug, 2020) and the presence of hypertrophied genal spines or complete loss of genal spines characterizes the group. Furthermore, phylogenetic analyses illustrated that Belinurina was a monophyletic superfamily traditionally thought to contain *Alanops* (Racheboeuf, Vannier & Anderson, 2002), *Anacontium* (Raymond, 1944), *Belinurus* (Pictet, 1846)*, Euproops* (Meek & Worthen, 1865), *Liomesaspis* (Raymond, 1944), and *Prolimulus* (Fritsch, 1899). Despite the interest in belinurids, an array of species described in the early 20th century require revision (see Lamsdell & McKenzie, 2015; Bicknell et al., 2019a: Bicknell, Lustri & Brougham, 2019; Bicknell & Pates, 2020; Bicknell & Smith, in press for revisions of similar historical material). Expanding on the recent pulse in the revision of such historically important species, we reevaluate *Prolimulus woodwardi* Fritsch, 1899. We present a phylogenetic analysis including *P. woodwardi* and the morphologically comparable *Stilpnocephalus pontebbanus* Selden, Simonetto & Marsiglio, 2019, as well as a geometric morphometric analysis of species within Belinurina. These analyses highlight the extreme morphologies exhibited by *Prolimulus* and its kin, suggesting the requirement for a clade to contain these species.

## Geologic and Stratigraphic Context

**Figure 1:**
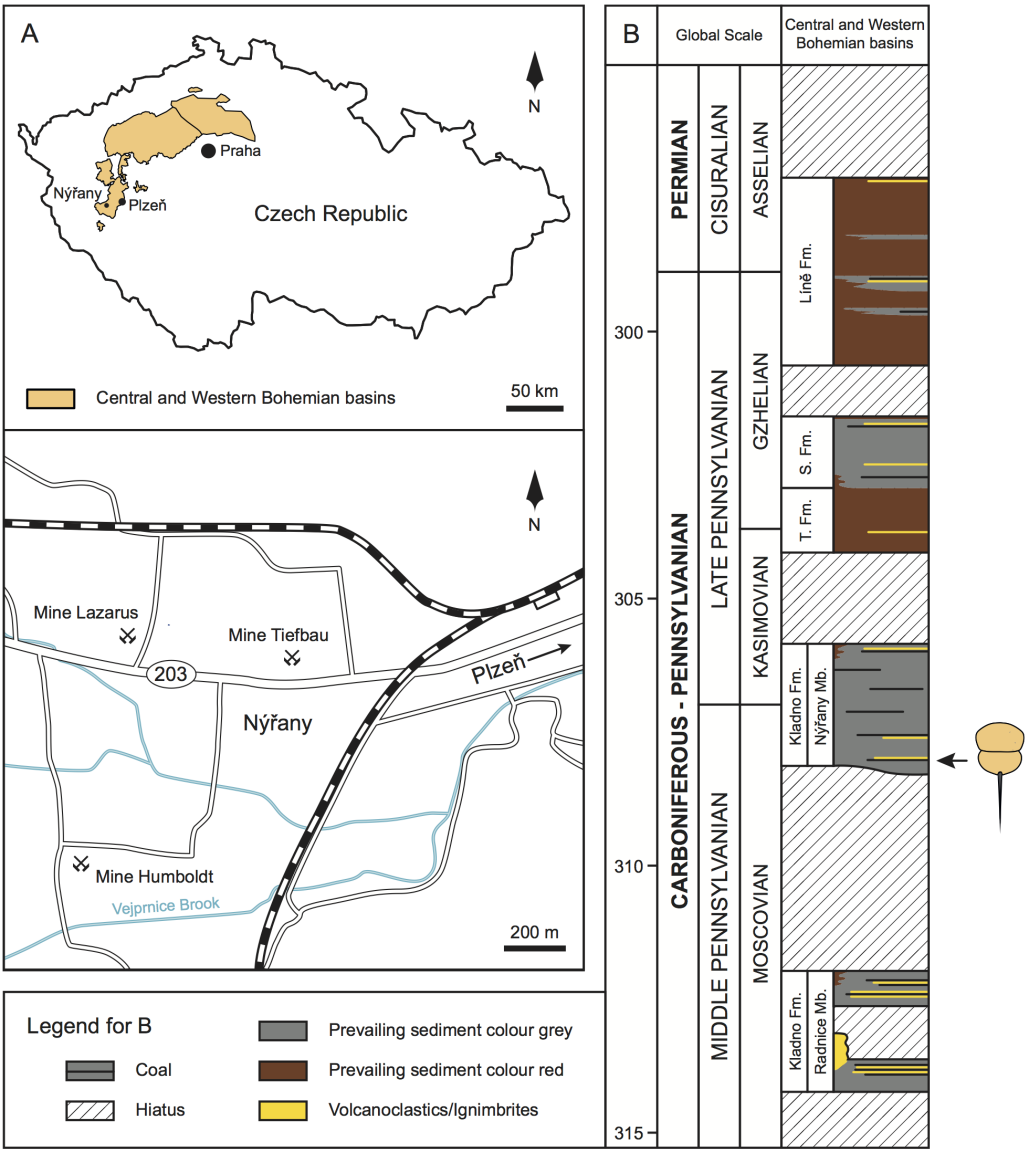
Geologic and stratigraphic context of the studied material. (A) Geographic location of the central and western Bohemian basins and location of the main historical mines in the Nýřany area. The studied material was collected from the Main Nýřany Coal, Humboldt Mine. (B) Chronostratigraphic position of the lithostratigraphic units of central and western Bohemian basins (modified from Opluštil et al., 2016). Arrow indicates the stratigraphic location of the studied material. Abbreviations: Fm., Formation; Mb., Member; S. Fm., Slaný Formation; T. Fm., Týnec Formation.

Upper Paleozoic continental strata in central and western Bohemia are formally subdivided into the Plzeň, Manětín, Žihle, Radnice, Kladno-Rakovník, and Mšeno-Roudnice basins (Pešek, 1994; Opluštil et al., 2013; Pešek, Sivek & Sivek, 2016; Fig. 1A). Sedimentary successions within these basins comprise of four formations—the Kladno Formation (composed of older Radnice and younger Nýřany members), that unconformably overlies the basement rocks; followed by the Týnec Formation, Slaný Formation, and terminated by the Líně Formation (Pešek, 1994; Opluštil et al., 2013; Fig. 1B).

The Nýřany Member of the Kladno Formation is composed of cyclically arranged, predominantly coarse- and medium-grained sediments of fluvial origin (Pešek, 1994; Opluštil, Martínek & Tasáryová, 2005). Fine-grained sediments of floodplain, palustrine, and lacustrine origin are also present (Opluštil, Martínek & Tasáryová, 2005). These cycles are usually terminated by thin coal seams (Pešek, 1994; Opluštil, Martínek & Tasáryová, 2005). From a palaeoenvironmental perspective, the Nýřany Member was deposited in a large alluvial plain with a braided river system with locally developed lakes, wetlands, and peat swamps (Opluštil, Martínek & Tasáryová, 2005), located in a nearly equatorial latitude (Krs, Krsová & Pruner, 1995). Recent U-Pb dating estimated that the Nýřany Member is between 308.3–305.9 ± 0.1 Ma, spanning the late Moscovian to early Kasimovian (Opluštil et al., 2016; Fig. 1B).

In the Plzeň Basin, the lower parts of the Nýřany Member contain the locally developed Main Nýřany Coal with intercalated beds of lacustrine sapropelic coal (Fritsch, 1883; Purkyně, 1899; Pešek, 1994; Štamberg & Zajíc, 2008). This sapropelic coal yielded diverse and exceptionally well-preserved fauna. Most of sapropelic coal fossils originated from the Humboldt Mine in Nýřany (near Plzeň, Fig. 1A) and represent various euarthropods (including *Prolimulus woodwardi*), acanthodians, chondrichthyans, dipnoans, actinopterygians, and early diverging tetrapods (Fritsch, 1883; Fritsch, 1902; Štamberg & Zajíc, 2008). After closure of the Nýřany and Třemošná coalfields, the sapropelic coal was unavailable for sampling, until a recent excavation (Bures & Tichavek, 2012).

## Material and Methods

### Systematic framework

We follow the systematic taxonomy of Lamsdell (2013), Lamsdell (2016), Lamsdell (2020a), Bicknell, Lustri & Brougham (2019), and Bicknell & Pates (2020) and anatomical terms presented in Selden & Siveter (1987), Haug & Rötzer (2018b), and Selden, Simonetto & Marsiglio (2019).

### Specimen photography

Museums where *Prolimulus woodwardi* specimens are housed were contacted and photographs of specimens were either requested from the collection managers or made by the authors, or colleagues. Most specimens were photographed with SLR cameras under normal light. Select specimens were submerged in alcohol prior to photography to enhance contrast; however, this could not be conducted for all specimens due to collection constraints.

### Phylogenetic analyses

The phylogenetic analysis was conducted to determine where *Prolimulus woodwardi* and the morphologically comparable *Stilpnocephalus pontebbanus* are located in tree space. These species were coded into the Bicknell, Lustri & Brougham (2019) matrix, derived from Lamsdell (2016). The analysis was performed under equally weighted parsimony in TNT 1.5 (Goloboff & Catalano, 2016) following Bicknell, Lustri & Brougham (2019) and Lamsdell (2016). Further, implied and equal weighted produced highly comparable trees. Five replications of a “New Technology” tree search was run using random sectorial searches, 1,000 iterations of the parsimony ratchet, 50 cycles of drifting and 5 rounds of tree fusing, holding a maximum of 10 trees per replication (Supplementary Information 1). All multistate characters were unordered (Lamsdell, 2016; Bicknell, Lustri & Brougham, 2019).

### Geometric morphometric methods

**Figure 2:**
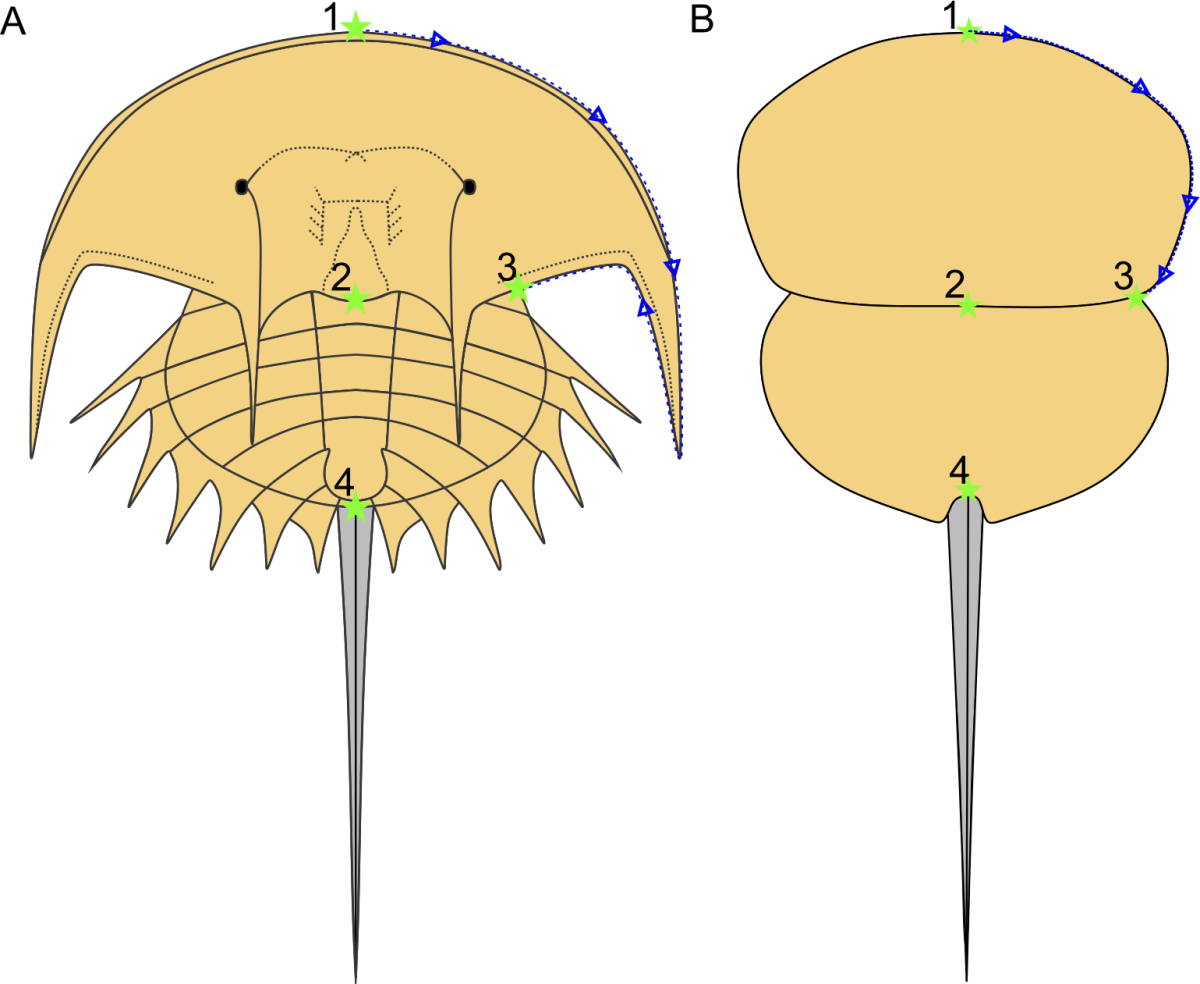
Approximate semilandmark trajectory (blue arrows and dotted line) and the landmarks used here. (A) Reconstruction of *Euproops danae* showing approximate landmark and semilandmark placement. (B) Reconstruction of *Prolimulus woodwardi* showing approximate landmark and semilandmark placement. Landmarks are described in Table 1.

Following Bicknell et al. (2019), a morphometric dataset of landmarks and semilandmarks from 91 specimens across 19 species was collected to explore Belinurina morphospace. Landmarking and semilandmarking was conducted using the Thin-Plate Spline (TPS) suite (http://life.bio.sunysb.edu/morph/index.html). The TPS file was constructed using tpsUtil64 (v.1.7). The TPS file was imported into tpsDig2 (v.2.26), which was used to place four landmarks across the prosoma and thoracetron and 40 semi-landmarks along the right prosomal shield (Fig. 2; Table 1). Semilandmarks were placed in a clockwise direction along the most anterior section of the prosomal shield, coinciding with the first landmark, ending at the third landmark: the most lateral prosomal-thoracetronic articulation point. Points were digitised as *xy* coordinates. When the right side was poorly preserved, the left side was used, and these data were mirrored. These data populated the TPS file (Supplementary Information 2). TPS file was imported into R. The ‘geomorph’ package (Adams & Otárola-Castillo, 2013) was used to conduct a Procrustes Superimposition and Principal Components Analysis (PCA) of the data (Data S3). Only the first two Principal Components (PCs) were considered as they explained 87% of the variation in the data. The examined species were representatives of *Alanops*, *Belinurus, Euproops, Liomesaspis,* and *Prolimulus*. We were unable to include *Anacontium* and *Stilpnocephalus* as opisthosomal sections are not known from these genera. We had initially used generic assignment of Bicknell & Pates (2020) for this analysis. However, during the course of peer review, Lamsdell (2020b) presented a revision of Xiphosurida and proposed that Belinurina consisted of 14 genera. To compare, contrast, and explore the distribution of these newly erected groups with the more conservative perspective of Bicknell & Pates (2020), we presented the distribution of genera suggested in Bicknell & Pates (2020) and Lamsdell (2020b).

**Table 1:** Description of landmarks. Landmarks used for the geometric morphometric analysis depicted in Fig. 2.

**Landmark number**	**Description of landmark**
Landmark 1	Anterior-most prosomal point along organismal sagittal line
Landmark 2	Distal-most prosomal point along organismal sagittal line
Landmark 3	Lateral-most section of prosomal-thoracetron articulation
Landmark 4	Thoracetron-telson articulation

## Systematic Palaeontology

**Table:** 

Euchelicerata sensu Weygoldt & Paulus, 1979
Xiphosurida sensu Latreille, 1802
Belinurina sensu Zittel & Eastman, 1913
Belinuridae sensu Zittel & Eastman, 1913
*Prolimulus*Fritsch, 1899

**Amended diagnosis:** Belinurid with a round prosoma that is slightly wider than long. No eyes, cardiac lobe, or ophthalmic ridges are present. Thoracetron is completely fused, without traces of segmentation, often showing a thoracetronic doublure. Thoracetron-telson articulation is ‘U’-shaped. Telson is keeled.

**Table:** 

**Figure 3:**
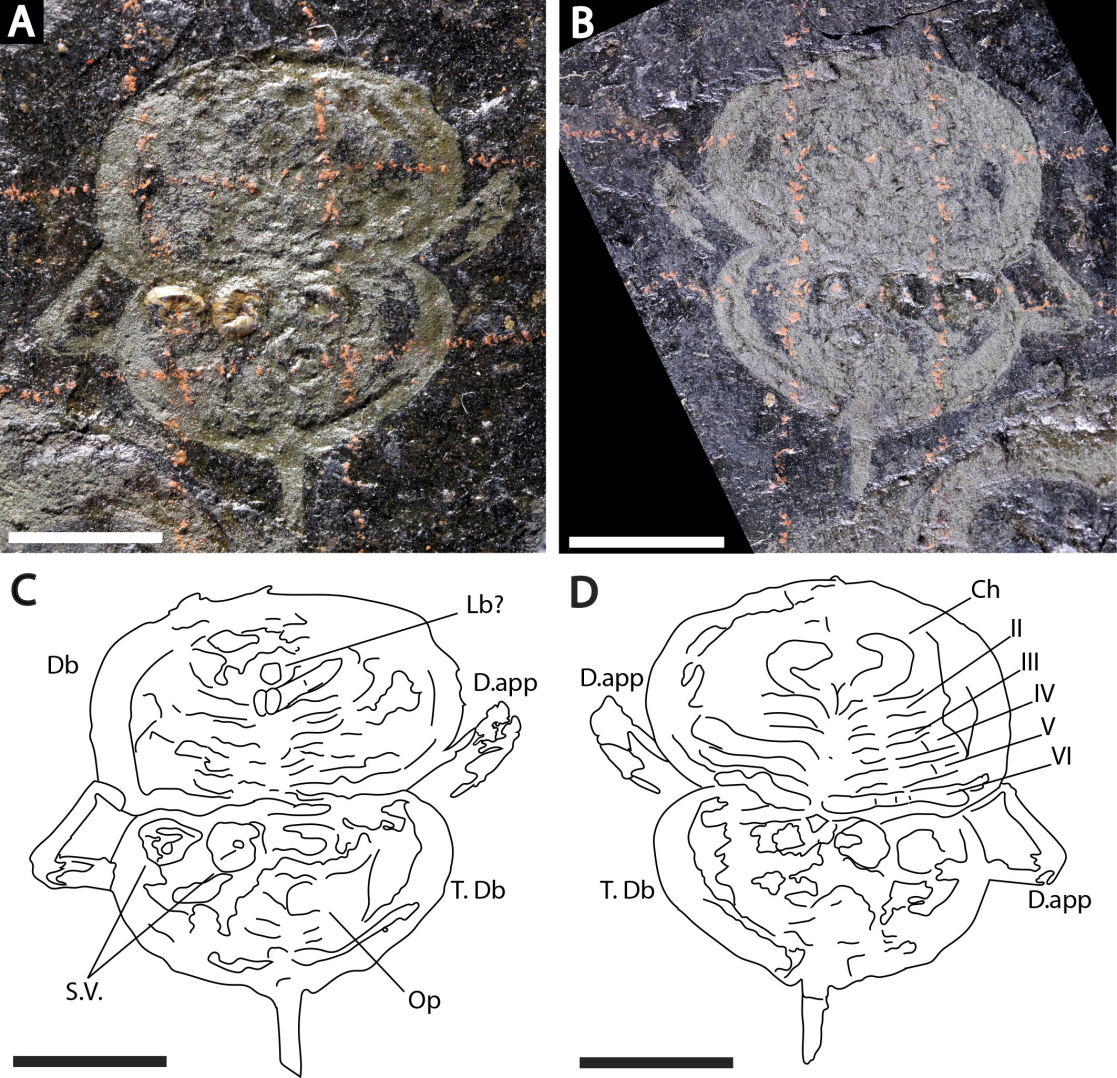
Holotype of *Prolimulus woodwardi* illustrating the general anatomy of the appendages. (A, C) NM Me 1031; part. (A) Complete specimen. (C) Interpretative drawing. (B, D) NM Me 1032; counterpart. (B) Complete specimen. (D) Interpretative drawing. Abbreviations: Ch: chelicera, D.app: disarticulated appendage, Db: prosomal doublure, II—VI: prosomal leg numbers, Lb: labium, Op: opercula, S.V.: *Spiroglyphus vorax*, T.Db: thoracetronic doublure. Scale bars: 10 mm. Image credit: Russell Bicknell.

**Figure 4:**
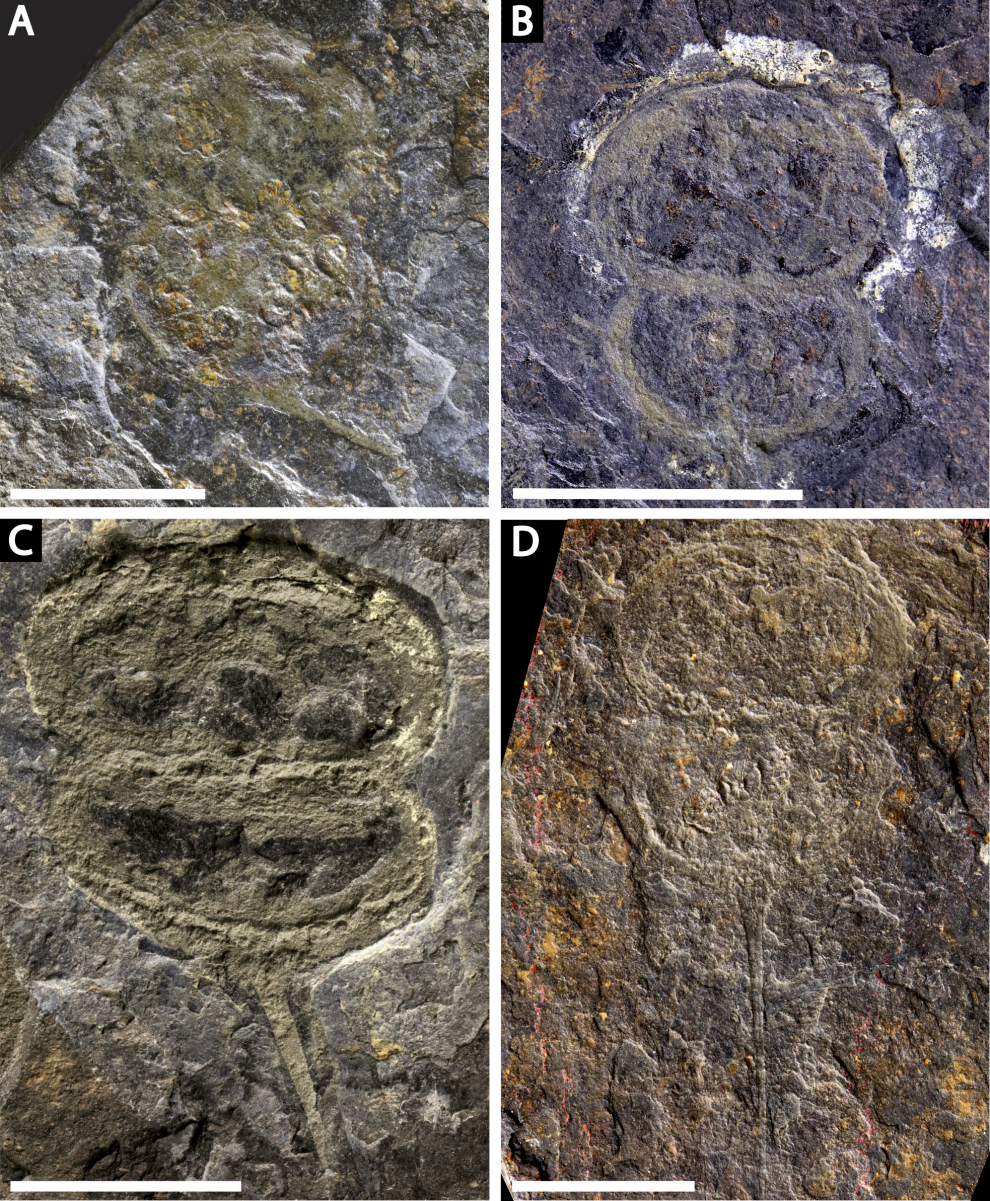
Four specimens of *Prolimulus woodwardi* from the National Museum of Prague Paleozoic Invertebrate collection. (A) NM Me 146. (B) NM Me 39. (C) NM Me 142. (D) NM Me 145. Scale bars: 10 mm. Image credit: Russell Bicknell.

**Figure 5:**
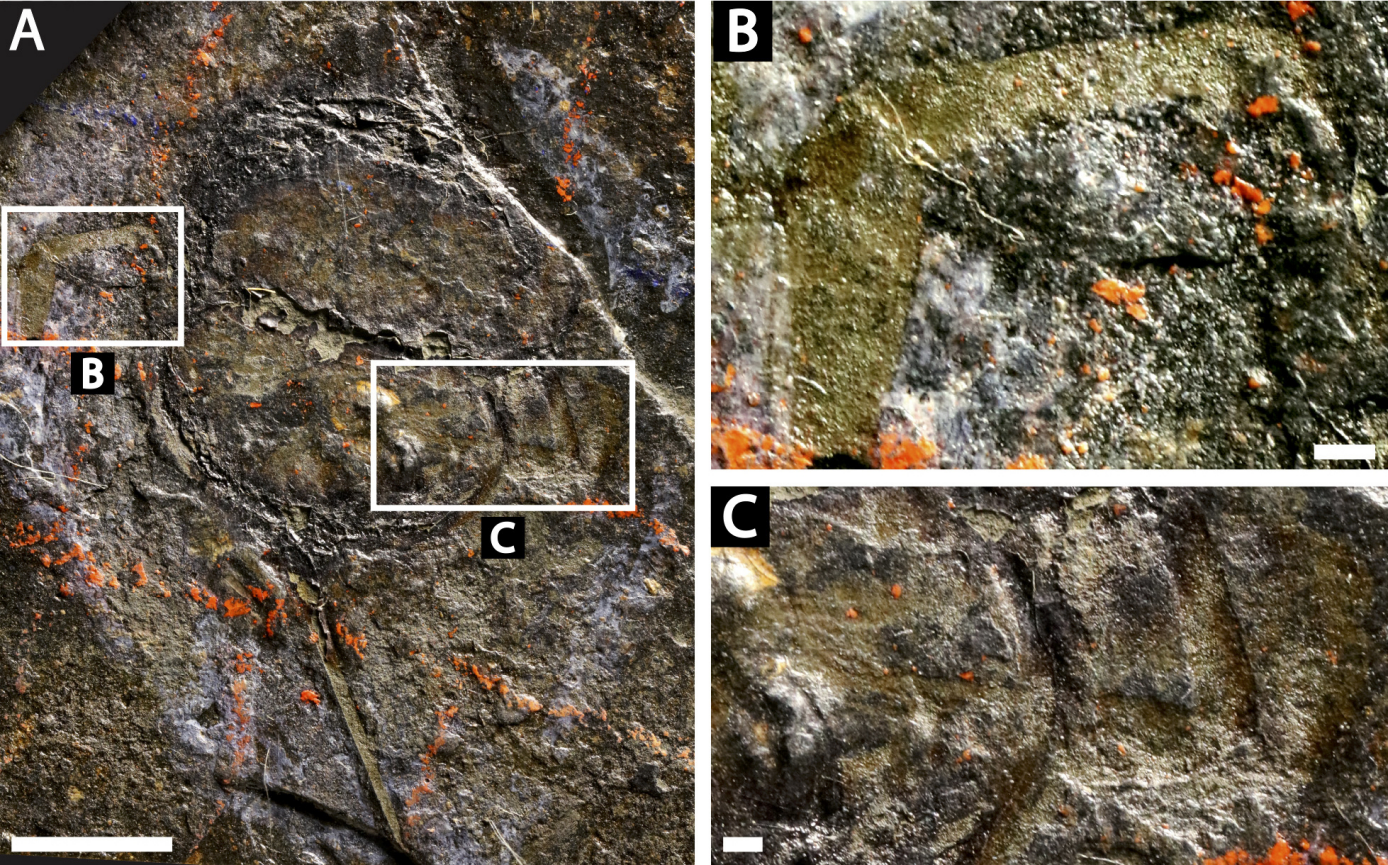
*Prolimulus woodwardi* specimen showing detailed appendicular features. (A, B, C) NM Me 1038. (A) Complete specimen. (B) Close up of left appendages. (C) Close up of right appendages. Scale bars: A: 10 mm, B, C: 1 mm. Image credit: Russell Bicknell.

**Figure 6:**
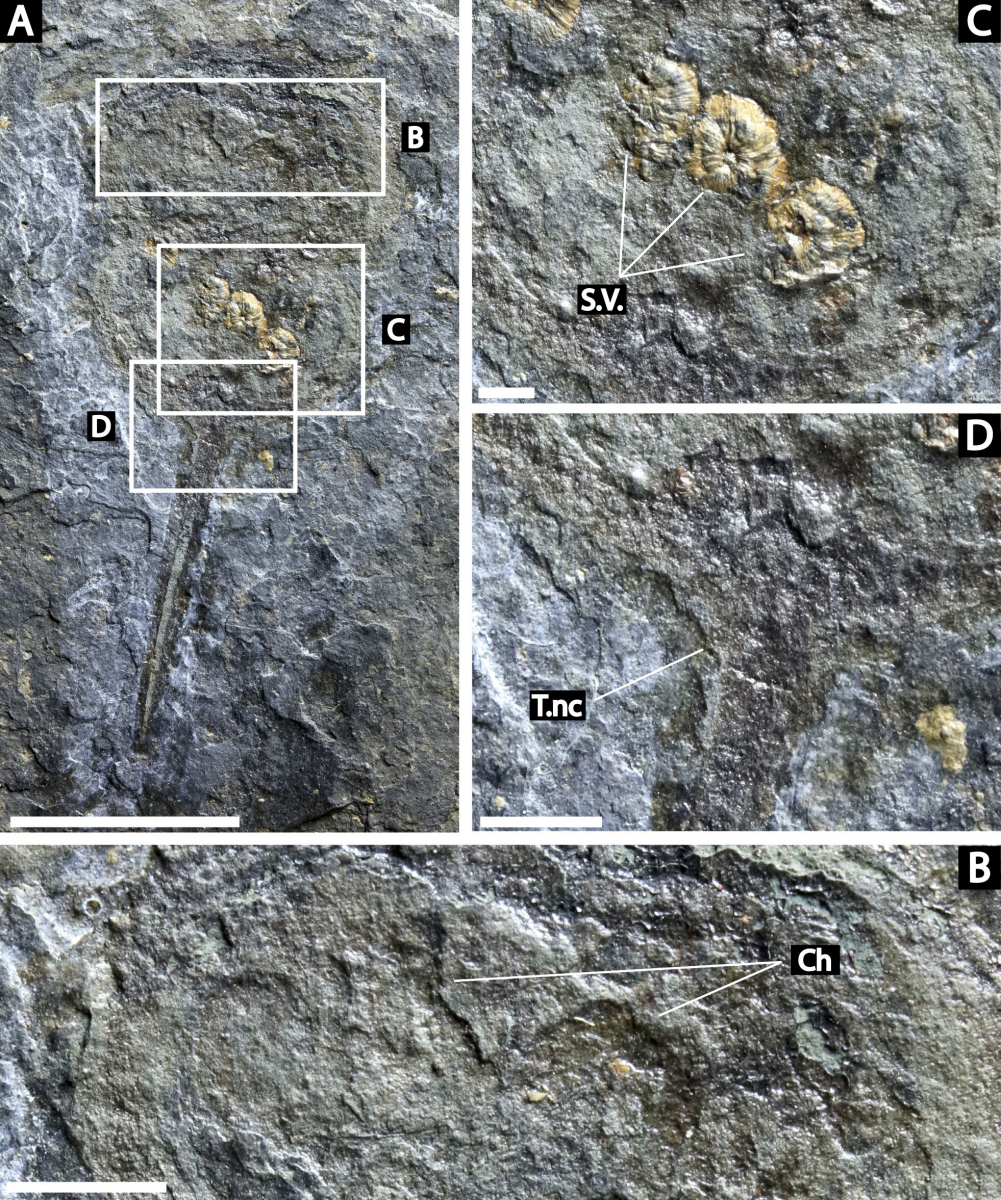
*Prolimulus woodwardi* specimen illustrating epibionts and thoracetron-telson articulation. (A, B, C) NM M 1045. (A) Complete specimen. (B) Close up on *Spiroglyphus vorax*. (C) Close up on telson notch. Abbreviations: Ch: chelicera, S.V.: *Spiroglyphus vorax*, T.nc: telson notch. Scale bars: A 10 mm; B 2 mm; C, D 1 mm. Image credit: Russell Bicknell.

**Figure 7:**
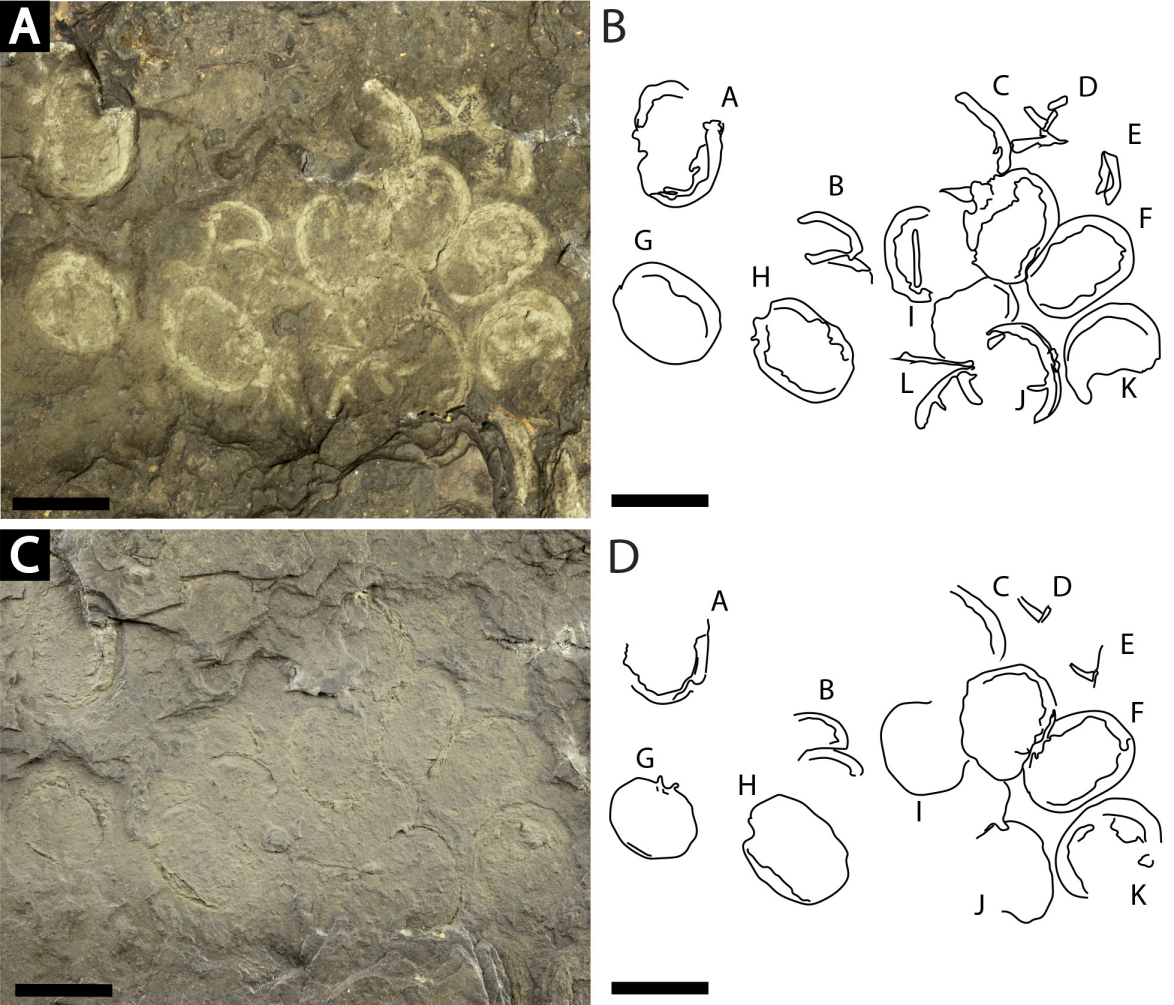
Slab of twelve *Prolimulus woodwardi* individuals recording possible gregarious behavior. NM Me 108. (A) Specimen photographed submerged in alcohol. (B) Interpretative drawing of (A). (C) Specimen photographed under natural light. (D) Interpretative drawing of (D). A—L indicate individual specimen designation. Scale bars: 10 mm. Image credit: Russell Bicknell.

**Figure 8:**
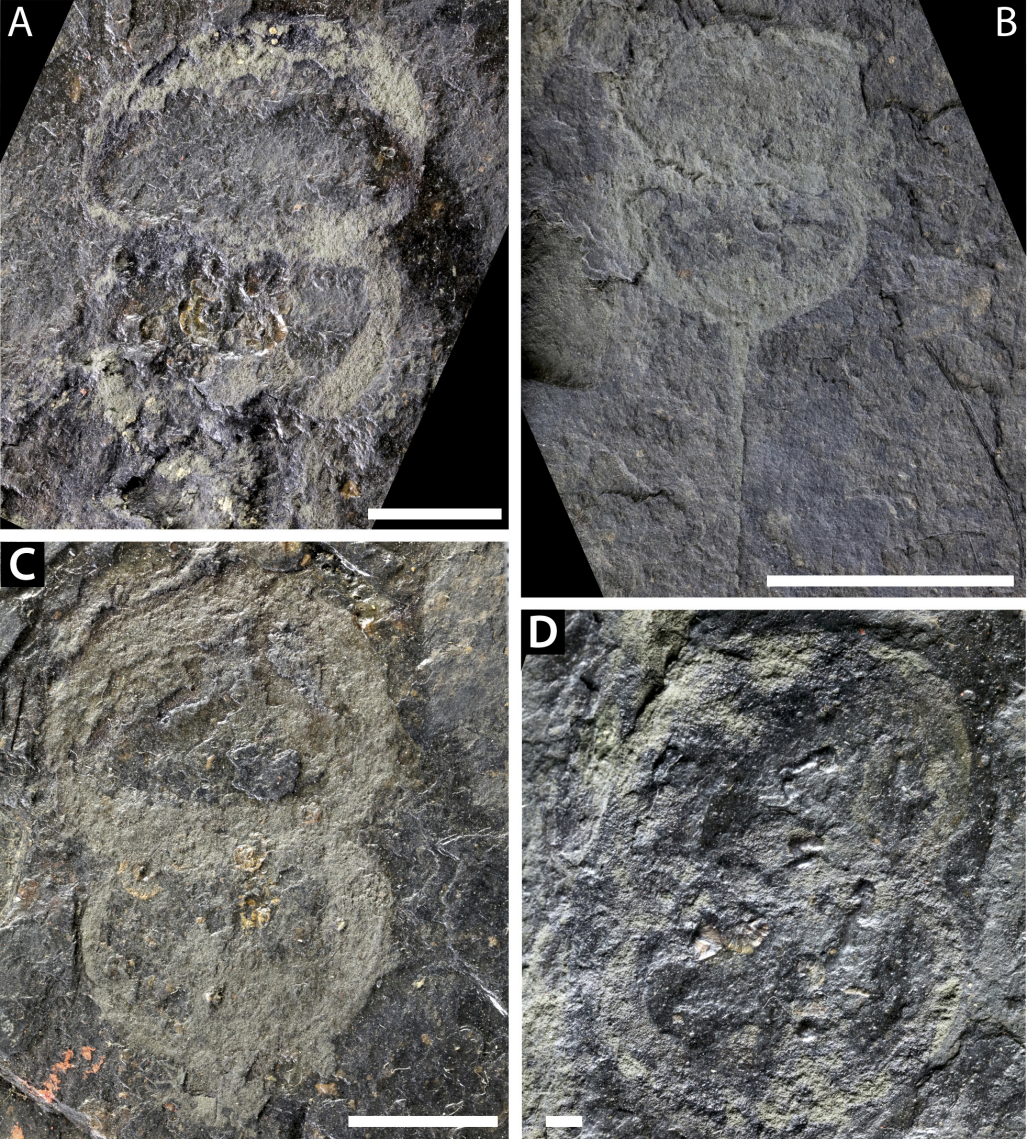
Further *Prolimulus woodwardi* specimens from the National Museum of Prague Paleozoic Invertebrate collection. (A) NM Me 141. (B) NM Me 109. (C) NM Me 139. (D) NM Me 143. Scale bars: A, B, C 10 mm; D 1 mm. Image credit: Russell Bicknell.

**Figure 9:**
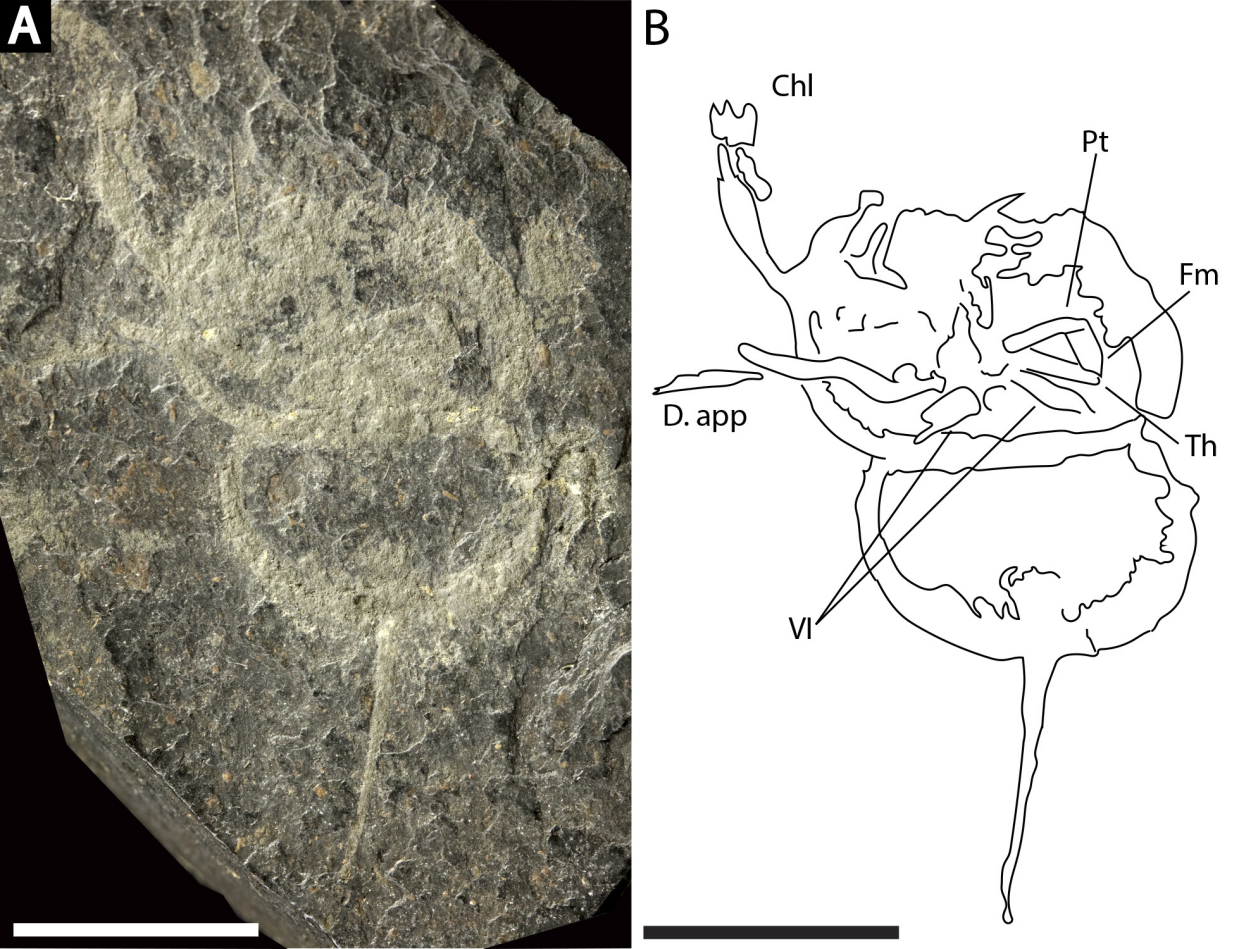
*Prolimulus woodwardi* specimen illustrating prosomal appendage morphology. (A) NM Me 140. (B) Interpretative drawing of specimen. Abbreviations: Chl: chelate podomeres, D.app: disarticulated appendage, VI: sixth prosomal appendage set, Pt: patella, Fm: femur, Th: trochanter. Scale bars: 10 mm. Image credit: Russell Bicknell.

**Figure 10:**
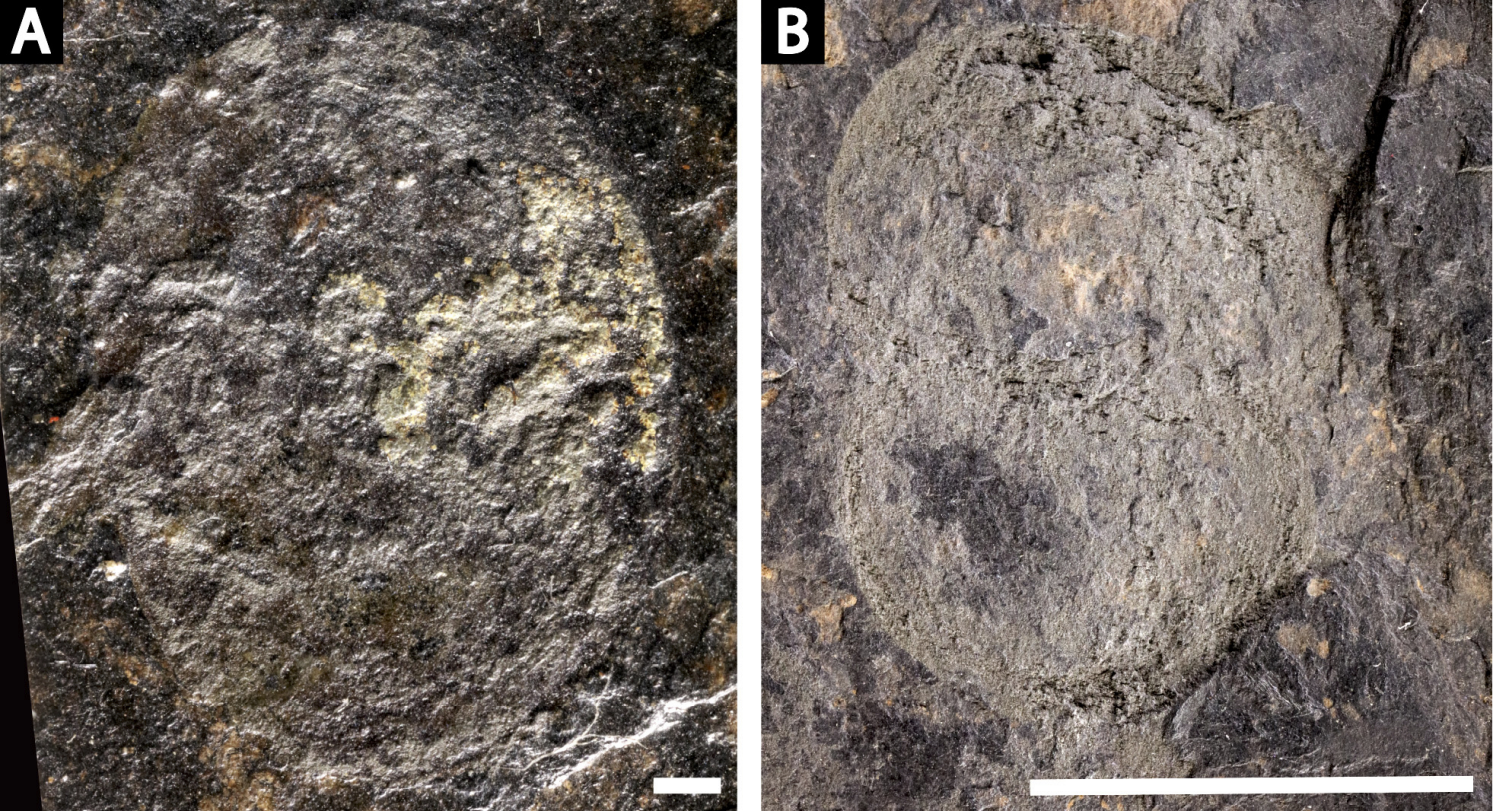
Poorly preserved *Prolimulus woodwardi* specimens from the National Museum of Prague Paleozoic Invertebrate collection. (A) NM Me 144. (B) NM M138. Scale bars: A: 1 mm; B: 10 mm. Image credit: Russell Bicknell.

**Figure 11:**
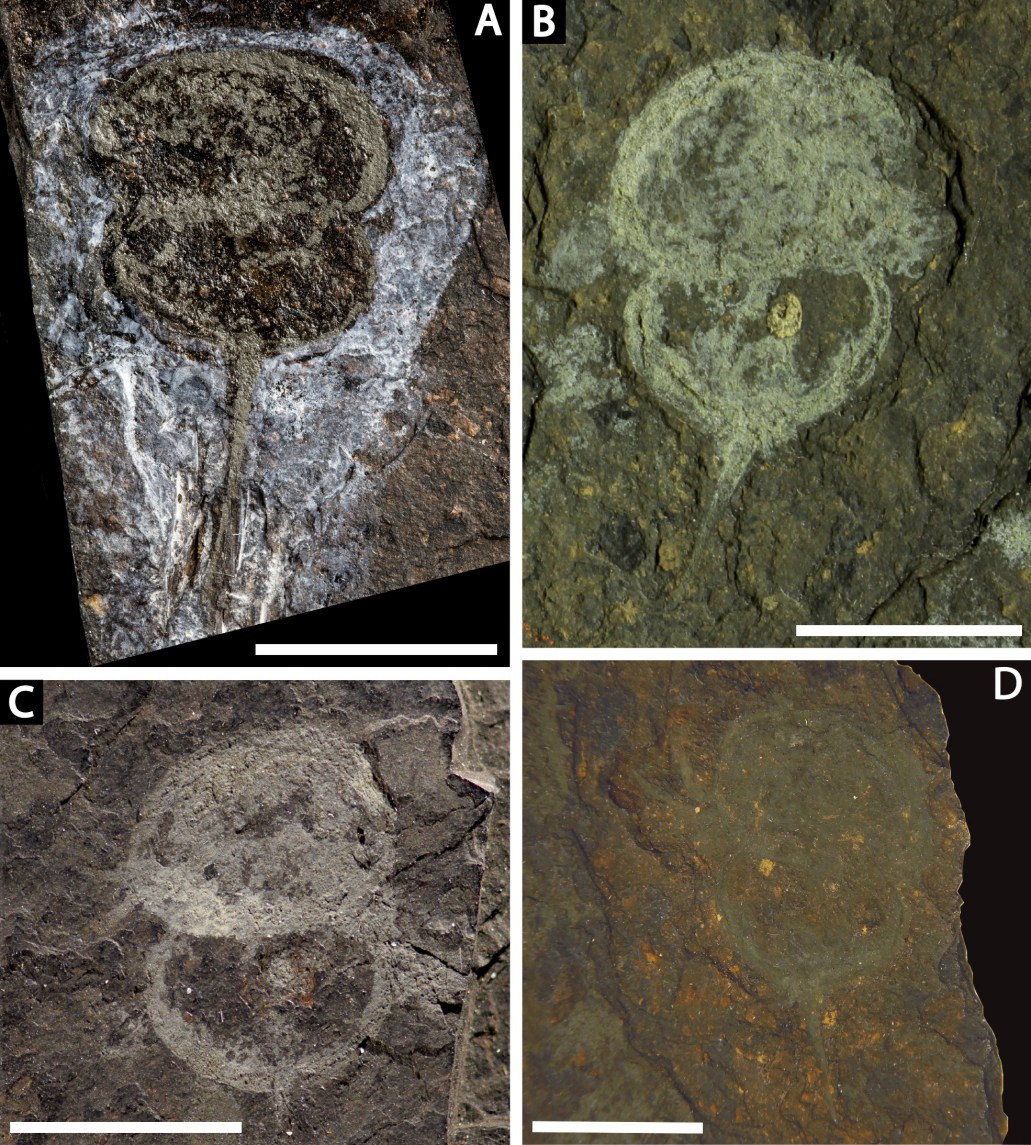
*Prolimulus woodwardi* specimens from the Museum für Naturkunde, Leibniz-Institut, the Museum of Comparative Zoology, and the Natural History Museum. (A) NHMUK PI In 18588; syntype. (B) NHMUK PI I 3395. (C) MCZ 109537. (D) MB.A. 1989. Scale bars: 10 mm. Image credit (A): Lucie Goodayle. (B, C): Stephen Pates. (D) Andreas Abele. Image in (A) reproduced from Bicknell & Pates (2020) under a CC BY 4.0 license.

**Figure 12:**
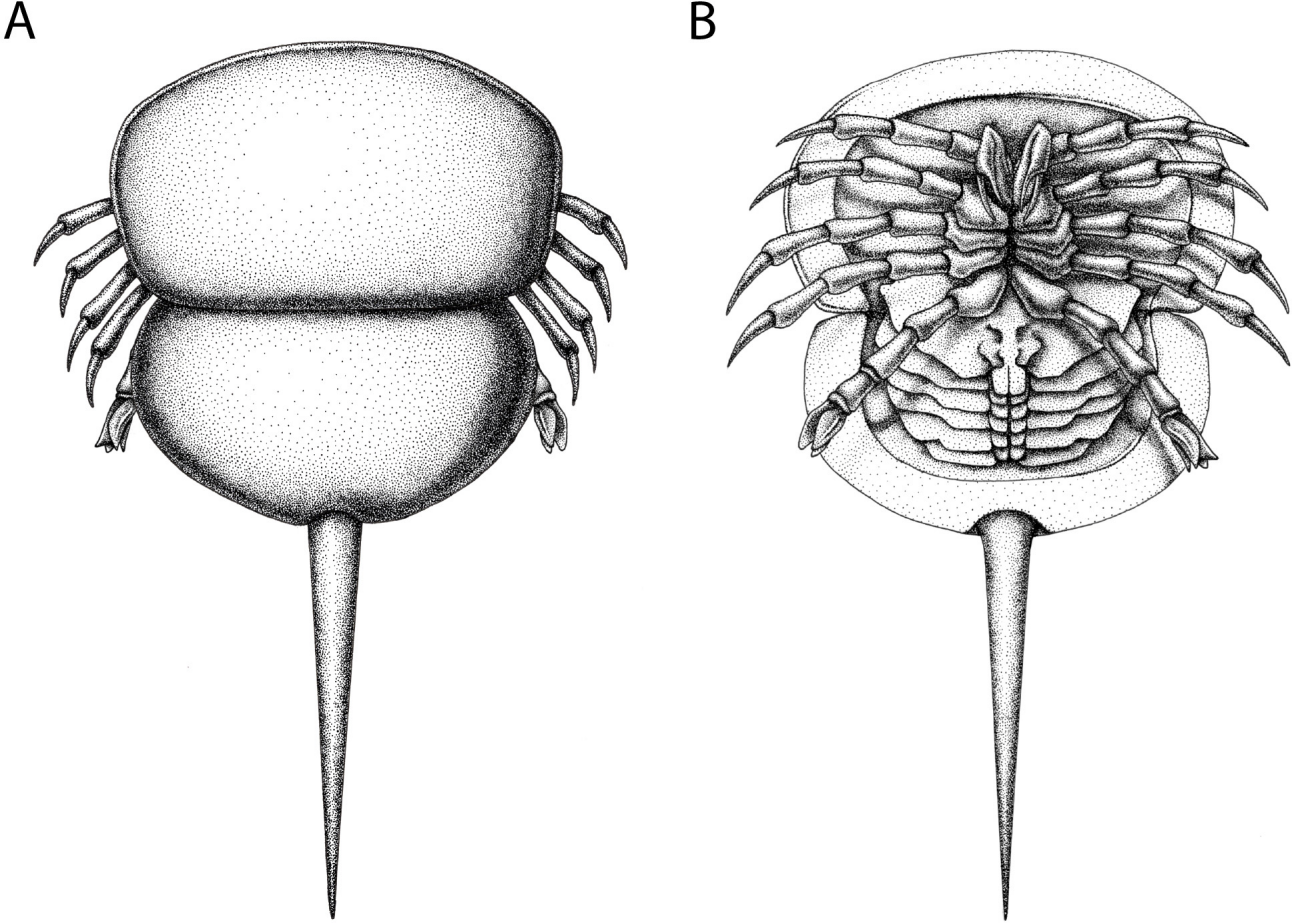
Reconstruction of *Prolimulus woodwardi*. (A) Dorsal anatomy based on the studied material. (B) Ventral anatomy: doublure and telson insertion based on studied material, appendages reflect details observed here and general belinurid anatomy. Reconstruction credited to Elissa Sorojsrisom.

*Prolimulus woodwardi* (Fritsch, 1899)
**Figs. 3–12**
1899 *Prolimulus woodwardi* Fritsch, p. 58
1902 *Prolimulus woodwardi* Fritsch, Fritsch p. 64
1938 *Prolimulus* Fritsch, Eller (1938, p. 153)
1944 *Prolimulus woodwardi* Fritsch, Raymond, p. 503
1948 *Prolimulus woodwardi* Fritsch, Branson (1948, p. 991)
1952 *Prolimulus* Fritsch, Størmer, p. 636
1955 *Prolimulus woodwardi* Fritsch, Prantl & Přibyl, pl. 2
1966 *Prolimulus* Fritsch, Strauch (1966, p. 271)
1975 *Prolimulus* Fritsch, Bergström (1975, p. 303)
1984 *Prolimulus woodwardi* Fritsch, Fisher (1984, fig. 2)
1990 *Prolimulus* Fritsch, Beall & Labandeira (1990, fig. 1)
1994 *Prolimulus* Fritsch, Rosa et al. (1994, fig. 8B)
1997 *Prolimulus woodwardi* Fritsch, Krawczyński, Filipiak & Gwoździewicz (1997, p. 1271)
2005 ?*Prolimulus* Fritsch, Crônier & Courville (2005, p. 128)
2016 *Prolimulus* Fritsch, Lamsdell, p. 188
2019 *Prolimulus* Fritsch, Selden et al., p. 335
2019 *Prolimulus* Fritsch, Bicknell & Pates, p. 1
2020 *Prolimulus woodwardi* Fritsch, Bicknell & Pates, figs. 21D–21F
2020b *Prolimulus woodwardi* Fritsch, Lamsdell, p. 17

**Holotype:** NM M Me 1031; NM M Me 1032

**Syntype:** NHMUK PI In 18588

**Referred material:** MB.A 1989; MCZ 109537; NM Me 39; NM Me 108; NM Me 109; NM Me 138; NM Me 139; NM Me 140; NM Me 141; NM Me 142; NM Me 143; NM Me 144; NM Me 145; NM Me 146; NM M 1038; NM M 1045; NHMUK PI I 3395.

**Locality, horizon, and age:** Nýřany (Humboldt Mine, active between 1865 –1902), Plzeň Basin; Main Nýřany Coal, Nýřany Member of the Kladno Formation; ∼307–308 Ma, late Moscovian, Pennsylvanian.

**Descriptions:** NM M 1031 and NM M 1032 (Fig. 3) are part and counterpart originally described and figured by Fritsch (1899), Fritsch (1902) and revised in Prantl & Pribyl (1955). They consist of an articulated prosoma, thoracetron, and telson, preserved as flattened impressions in ventral view. Prosoma is round, slightly wider than long: 20 mm wide and 11.9 mm long. The prosomal doublure is preserved and has a maximum width of 1.9 mm. No genal spines, lateral compound eyes, cardiac lobe, or ophthalmic ridges noted. Possible traces of a labium are preserved in NM Me 1031 (Figs. 3A, 3C). Appendages are preserved and mainly visible on NM Me 1032. Chelicera are present, but chelate podomeres are not preserved (Figs. 3B, 3D). Proximal sections of walking legs are preserved as slight imprints. Furthermore, podomeres of the sixth appendages are noted (Fig. 3). Third and fourth podomeres of two disarticulated appendages preserved outside prosoma. Thoracetron lacks tergal expression, is round and slightly smaller than prosoma: 18.5 mm wide and 10 mm long. No lateral spines noted. A prominent margin—likely thoracetronic doublure—preserved, is wide 2.1 mm (Fig. 3). Poorly preserved opercula are present on posterocentral thoracetron (Figs. 3A, 3C). Telson articulates with thoracetron along on posterior thoracetron margin. Telson 4.7 mm long and fragmentally preserved. Four *Spiroglyphus vorax* Fritsch, 1895 specimens are attached to thoracetron.

**NM** **Me 146 (Fig. 4A)**: Articulated prosoma, thoracetron and partial telson, preserved as flattened impression in ventral view. Prosoma round, slightly wider than long: 11.5 mm wide and 8.5 mm long. No prosomal doublure, genal spines, lateral compound eyes, cardiac lobe, appendages, or ophthalmic ridges noted. Thoracetron lacks tergal expression, is round, and slightly smaller than prosoma: 10.9 mm wide and 7 mm long. No lateral spines are noted. Thoracetron-telson articulation unclear, but occurs on posterior thoracetron margin. Telson partly preserved and 8.2 mm long. Two specimens of *Spiroglyphus vorax* attached to prosoma, three to prosoma-thoracetron border, and ten to thoracetron.

**NM** **Me 39 (Fig. 4B):** Articulated prosoma and thoracetron, preserved as a flattened impression in ventral view. Prosoma round, slightly wider than long: 10.5 mm wide and 7.5 mm long with a pronounced prosomal doublure. No genal spines, lateral compound eyes, cardiac lobe, appendages, or ophthalmic ridges noted. Thoracetron lacks tergal expression, is round, and slightly smaller than the prosoma; 9.3 mm wide and 5.6 mm long. No lateral spines noted.

**NM** **Me 142 (Fig. 4C):** Articulated prosoma, thoracetron, and partial telson, preserved as a mostly flattened impression in ventral view. Limited relief noted in posterior prosoma, prosoma-thoracetron articulation, and anterior thoracetron. Prosoma round, slightly wider than long: 12.6 mm wide and 6.8 mm long, with a pronounced prosomal doublure. No genal spines, lateral compound eyes, cardiac lobe, or ophthalmic ridges noted. Chelicera, the left set of chelate podomeres, and the proximal sections of walking legs preserved. Thoracetron lacks tergal expression, is round, and slightly larger than prosoma: 11.5 mm wide and 5.9 mm long. Pronounced thoracetronic doublure noted, 1.7 mm wide. No lateral spines noted. Thoracetron-telson articulation unclear, but occurs on posterior thoracetron margin. Telson partly preserved, 6.7 mm long.

**NM** **Me 145 (Fig. 4D):** Articulated prosoma, thoracetron, and telson, preserved as flattened impression in ventral view. Prosoma round, slightly wider than long: 13.3 mm wide and 8 mm long. No prosomal doublure, genal spines, lateral compound eyes, cardiac lobe, or ophthalmic ridges noted. No appendages are preserved. Thoracetron lacks tergal expression, is round, and slightly smaller than prosoma: 11.8 mm wide and 8.2 mm long. No lateral spines noted. Telson articulates with posterior thoracetron margin. Telson completely preserved and 15 mm long. Five *Spiroglyphus vorax* specimens attached to thoracetron.

**NM** **M 1038 (Fig. 5):** Articulated prosoma, thoracetron, and partial telson, preserved as a flattened impression in ventral view. Prosoma round, slightly wider than long: 19 mm wide and 10.8 mm long. No prosomal doublure, genal spines, lateral compound eyes, cardiac lobe, or ophthalmic ridges noted. One disarticulated appendage preserved on left prosomal side (Fig. 5B). Thoracetron lacks tergal expression, is round, and slightly smaller than the prosoma: 17.5 mm wide and 10.3 mm long. No lateral spines noted. Thoracetron-telson articulation unclear, but occurs on posterior thoracetron margin. Telson partly preserved and 17.1 mm long. One *Spiroglyphus vorax* specimen attached to thoracetron.

**NM** **M 1045 (Fig. 6):** Articulated prosoma, thoracetron, and telson, preserved as a flattened impression in ventral view. Prosoma round, slightly wider than long: 11.6 mm wide and 7.5 mm long. No prosomal doublure, genal spines, lateral compound eyes, cardiac lobe, or ophthalmic ridges noted. Chelicera are only preserved appendages (Fig. 6B). Thoracetron lacks tergal expression, is round, and slightly smaller than prosoma: 10.5 mm wide and 7.5 mm long. No lateral spines noted. Thoracetron-telson articulation is a ‘U’-shaped indentation in posterior thoracetron margin (Fig. 6C). Telson completely preserved, 13 mm long, and keeled. Four *Spiroglyphus vorax* specimens attached to thoracetron and all show growth lines (Fig. 6C).

**NM** **Me 108 (Fig. 7):** Twelve individuals preserved on a slab. Mean prosomal size is 11.5 mm wide and 8.4 mm long, mean thoracetron size is 10.5 mm wide and 7.4 mm long. Two individuals (F and I) are articulated. In both cases, prosomal sections rotated relative to thoracetron. Both preserve prosomal and thoracetronic doublures. No genal spines, lateral compound eyes, cardiac lobe, or ophthalmic ridges noted for either specimen. Thoracetrons lack tergal expression and lateral spines. Individual I preserved telson insertion (Fig. 7). Four individuals (C, D, E, and L) are thoracetron and telson fragments. Five individuals (A, G, H, J, and K) likely represent disarticulated prosomal sections. Individual G shows a possible appendage pair on anterior prosomal edge. Individual B is fragmentary and may represent a disarticulated prosoma and thoracetron, or parts of different individuals. Cluster lacks any orientation and evidence of epibionts.

**NM** **Me 141 (Fig. 8A)**: Articulated prosoma and thoracetron, poorly preserved as a flattened impression in ventral view. Prosoma round, slightly wider than long: 19.4 mm wide and 11 mm long. No prosomal doublure, genal spines, lateral compound eyes, cardiac lobe, appendages, or ophthalmic ridges noted. Only right side of thoracetron preserved. Thoracetron lacks tergal expression and lateral spines. Five *Spiroglyphus vorax* specimens attached to thoracetron.

**NM** **Me 109 (Fig. 8B):** Articulated prosoma, thoracetron, and partial telson, preserved as a flattened impression in ventral view. Prosoma round, slightly wider than long: 10.3 mm wide and 5.9 mm long. No prosomal doublure, genal spines, lateral compound eyes, cardiac lobe, appendages, or ophthalmic ridges noted. Thoracetron lacks tergal expression, is round, and slightly smaller than prosoma: 9.3 mm wide and 5 mm long. No lateral spines noted. Thoracetron-telson articulation unclear, but occurs at posterior thoracetron margin. Telson partly preserved, 11 mm long. Two *Spiroglyphus vorax* specimens attached to thoracetron.

**NM** **Me 139 (Fig. 8C):** Articulated prosoma, thoracetron, and partial telson, poorly preserved as a flattened impression in ventral view. Prosoma round, slightly wider than long: 20 mm wide and 15 mm long, and preserves pronounced prosomal doublure. No genal spines, lateral compound eyes, a cardiac lobe, appendages, or ophthalmic ridges noted. Thoracetron lacks tergal expression, is round, and slightly smaller than prosoma: 17 mm wide and 12 mm long. No lateral spines noted. Thoracetron-telson articulation unclear, but occurs at posterior thoracetron margin. Telson fragmentary and 4.3 mm long. Three *Spiroglyphus vorax* specimens attached to thoracetron.

**NM** **Me 143 (Fig. 8D)**: Articulated prosoma and thoracetron, preserved as a flattened impression in ventral view. Prosoma round, slightly wider than long: 10 mm wide and 6.7 mm long. No prosomal doublure, genal spines, lateral compound eyes, cardiac lobe, or ophthalmic ridges noted. Thoracetron lacks tergal expression, is round, and slightly smaller than prosoma: and 9.6 mm wide and 6.4 mm long. No lateral spines noted. Four *Spiroglyphus vorax* specimens attached to thoracetron (Fig. 8D).

**NM** **Me 140 (Fig. 9):** Articulated prosoma, thoracetron, and telson, preserved as a flattened impression in ventral view. Prosoma round, slightly wider than long: 14.3 mm wide and nine mm long. Prosomal doublure present. No genal spines, lateral compound eyes, cardiac lobe, or ophthalmic ridges noted. Two disarticulated prosomal appendages are preserved outside left prosomal side. Anterior appendage consists of at least a trochanter, femur, and patella, while posterior appendage possesses possible apotele and pretarsus (Fig. 9B). Two appendage sets preserved exclusively within prosomal shield. The fifth appendage on right side preserves fully articulated femoral, patellar, and trochanteral sections (Fig. 9B). Coxal sections of the sixth appendage pair also noted (Fig. 9B). Thoracetron lacks tergal expression, is round, and slightly smaller than prosoma: 11.3 mm wide and 6.5 mm long. No lateral spines noted. Thoracetron-telson articulation unclear, but occurs at posterior thoracetron margin. Telson fragmentary, 9.6 mm long.

**NM** **Me 144 (Fig. 10A):** Articulated prosoma and thoracetron, poorly preserved as a flattened impression in ventral view. Prosoma round, slightly wider than long: 8.8 mm wide and 6.5 mm long. No prosomal doublure, genal spines, lateral compound eyes, cardiac lobe, ophthalmic ridges, or appendages noted. Thoracetron lacks tergal expression, is round, and slightly smaller than prosoma: 7.7 mm wide and 7.5 mm long. No lateral spines noted.

**NM** **Me 138 (Fig. 10B):** Articulated prosoma and thoracetron, poorly preserved as a flattened impression in ventral view. Prosoma round, slightly wider than long: 10.4 mm wide and 6.9 mm long. No prosomal doublure, genal spines, lateral compound eyes, cardiac lobe, ophthalmic ridges, or appendages noted. Thoracetron lacks tergal expression, is round, and slightly smaller than prosoma: 8.8 mm wide and 6.7 mm long. No lateral spines noted.

**NHMUK PI In 18588; syntype (Fig. 11A):** Articulated prosoma, thoracetron and telson, preserved as a flattened impression in ventral view. Prosoma round, slightly wider than long: 14 mm wide and 7 mm long. No prosomal doublure, genal spines, lateral compound eyes, cardiac lobe, ophthalmic ridges, or appendages noted. Thoracetron lacks tergal expression, is round, and slightly smaller than prosoma: 13.2 mm wide and 6.7 mm long. No lateral spines noted. Thoracetron-telson articulation unclear, but occurs at posterior thoracetron margin. Telson fragmentary, 12.5 mm long.

**NHMUK PI I 3395 (Fig. 11B)**: Articulated prosoma, thoracetron and telson, preserved as a flattened impression in ventral view. Prosoma round, slightly wider than long: 15.1 mm wide and 10 mm long. No prosomal doublure, genal spines, lateral compound eyes, cardiac lobe, ophthalmic ridges, or appendages noted. Thoracetron lacks tergal expression, is round, and slightly smaller than prosoma: 13.1 mm wide and eight mm long. No lateral spines are. Thoracetron-telson articulation unclear, but occurs at posterior thoracetron margin. Telson fragmentary, 9.5 mm long.

**MCZ 109537 (Fig. 11C):** Articulated prosoma, thoracetron and partial telson, preserved as a flattened impression in ventral view. Prosoma round, slightly wider than long: 10.8 mm wide and 8 mm long. No prosomal doublure, genal spines, lateral compound eyes, cardiac lobe, or ophthalmic ridges noted. Proximal sections of prosomal appendages are preserved outside prosoma. Thoracetron lacks tergal expression, is round, and slightly smaller than prosoma: 10 mm wide and 7 mm long. No lateral spines noted. Thoracetron-telson articulation unclear, but occurs at posterior thoracetron margin. Telson fragmentary, only articulation point preserved.

**MBA. 1989 (Fig. 11D)** Articulated prosoma, thoracetron and telson, preserved as a flattened impression in ventral view. Prosoma round, slightly wider than long: 13.9 mm wide and 9.9 mm long. No prosomal doublure, genal spines, lateral compound eyes, cardiac lobe, or ophthalmic ridges noted. A disarticulated appendage preserved on left side of prosoma. Thoracetron lacks tergal expression, is round, and slightly smaller than prosoma: 11.7 mm wide and 7.9 mm long. No lateral spines noted. Thoracetron-telson articulation unclear, but occurs at posterior thoracetron margin. Telson fragmentary, 6.5 mm long.

**Figure 13:**
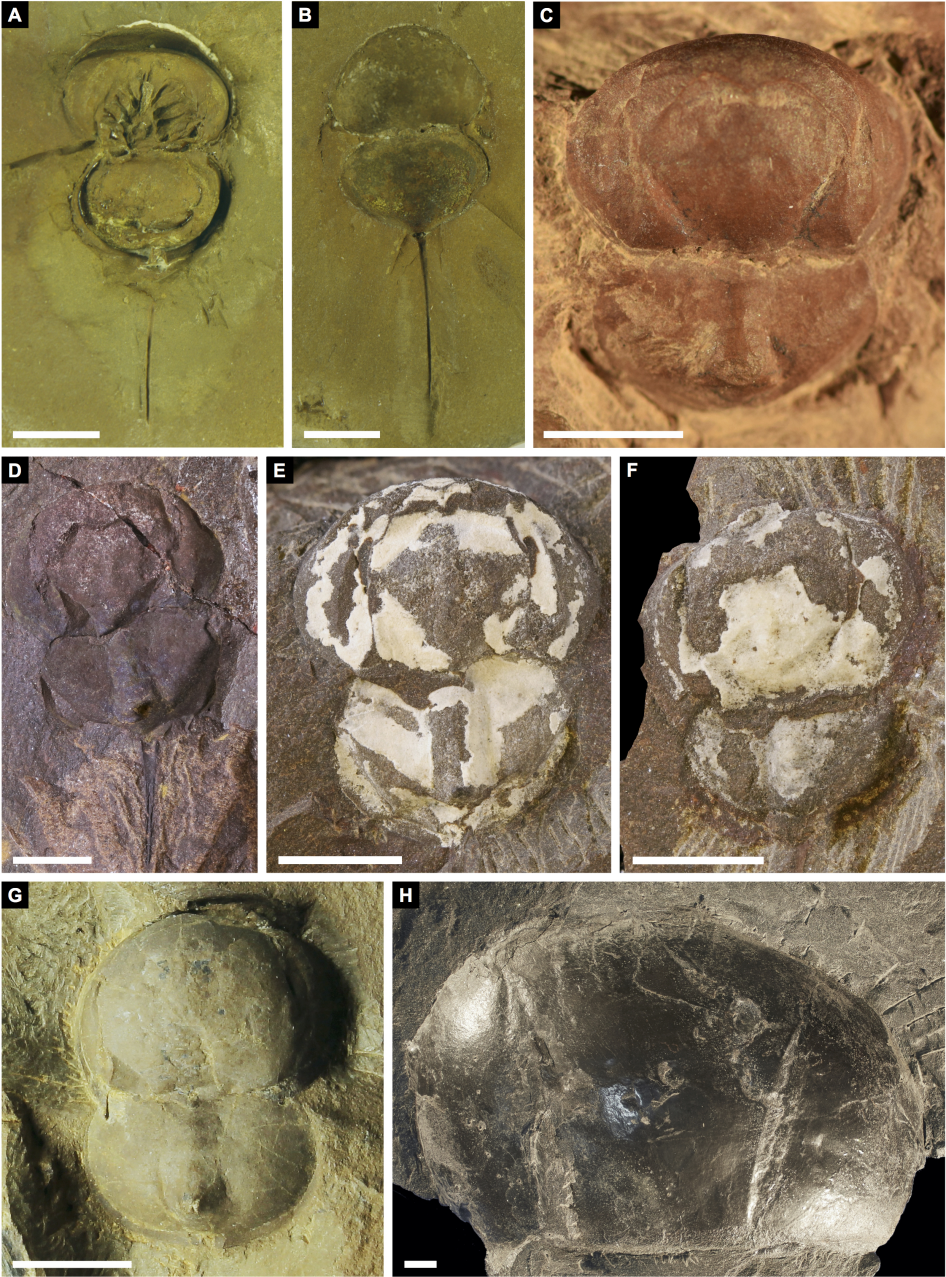
Other belinurids without genal spines. (A, B) *Alanops magnifica* from the Pennsylvanian (Stephanian)-aged Montceau-les-Mines Konservat-Lagerstätte, Great Seams Formation, France. (A) MNHN SOT001784, paratype. (B) MNHN SOT002154, paratype. (C—F) *Liomesaspis laevis* from the Pennsylvanian (Moscovian)-aged Mazon Creek *Konservat–Lagerstätte*, Carbondale Formation, USA. (C) MCZ 109536, holotype. (D) YPM IP 16913, paratype. (E) YMP IP 168041 (F) YMP IP 168053. (G) ?*Liomesaspis birtwelli* from the Pennsylvanian (Duckmantian)-aged Pennine Middle Coal Measures Formation, England, UK. NHMUK I 13882. (H) *Stilpnocephalus pontebbanus* from the Pennsylvanian (Kasimovian)-aged Meledis Formation, Friuli, MPT 18062301. Scale bars: 5 mm. Photo credit: (A, B) Dominique Chabard; (C—F) Russell Bicknell; (G) Stephen Pates; (H) Paul Selden.

**Figure 14:**
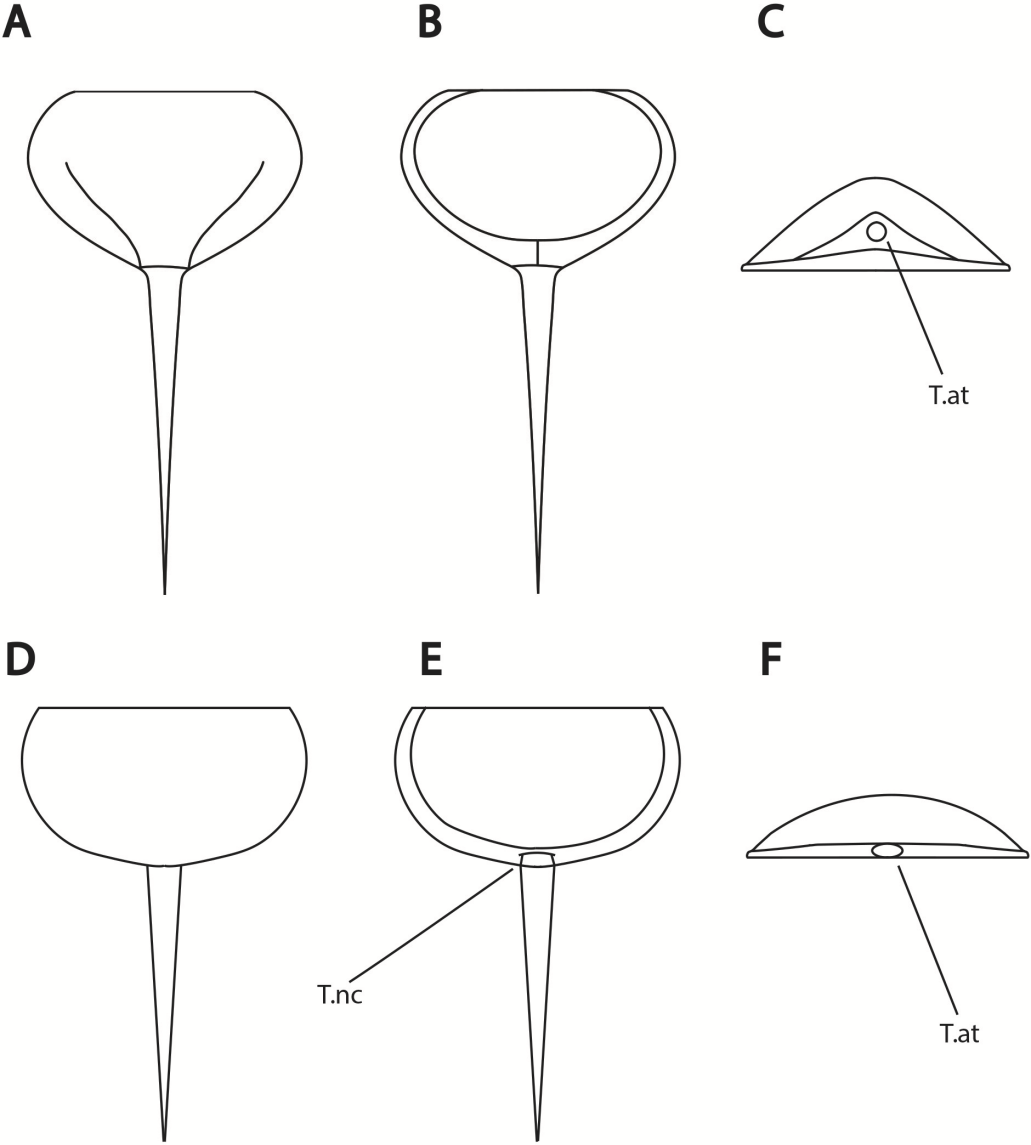
Differences in telson articulation between *Alanops magnificus* and *Prolimulus woodwardi*. (A–C) Schematic reconstruction of *Alanops magnificus* thoracetron and telson (modified from Racheboeuf, Vannier & Anderson, 2002). (A) Dorsal view. (B) Ventral view. (C) Posterior view. (D–F) Schematic reconstruction of *Prolimulus woodwardi* thoracetron and telson. (D) Dorsal view. (E) Ventral view. (F) Posterior view. Abbreviations: T.at: telson attachment, T.nc: telson notch.

**Remarks:** The prosoma and thoracetron shape of *Prolimulus woodwardi* is morphologically comparable to other belinurids without genal spines (Figs. 12 and 13). However, *P. woodwardi* has a unique telson insertion morphology. In *P. woodwardi* the insertion is a ‘U’-shaped indentation in the thoracetron, while *Alanops* and *Liomesaspis* lack this feature (Fig. 14). Furthermore, *Alanops* and *Liomesaspis* (Fig. 13) possess a ‘thoracetronic boss’, a bulge present on thoracetron over the insert of the telson. In *P. woodwardi* there is no evidence of this morphology. Finally, *Stilpnocephalus* has two notable grooves along the prosoma, not observed in *P. woodwardi*.

**Figure 15:**
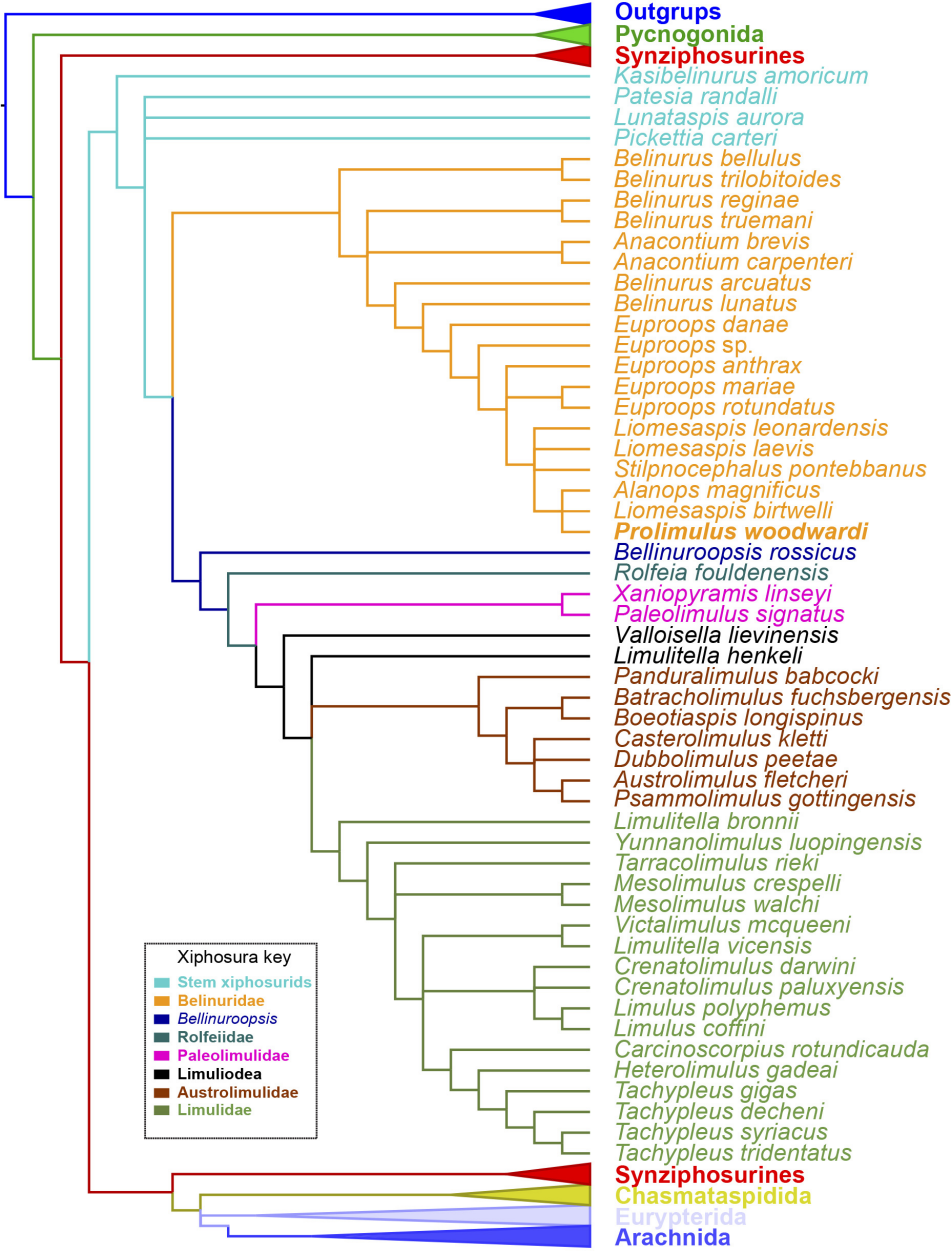
Results of the phylogenetic analysis. Strict consensus of the three trees produced by analyzing Supplementary Information 1. *Prolimulus woodwardi* is presented in bold. Topology of the outgroups, Synziphosura, Chasmataspidida, Eurypterida, and Arachnida collapsed as they are not considered here and are unchanged from other studies that used this dataset.

## Results

### Phylogenetic results

The phylogenetic analysis produced 3 trees of length 746. The strict consensus tree resultant from these trees have a comparable topology to other publications that have used the same matrix (Lamsdell, 2016; Bicknell, Lustri & Brougham, 2019; Bicknell, Naugolnykh & Brougham, 2020; Bicknell & Pates, 2019; Fig. 15). The main difference is the grouping of species within Belinurina. *Prolimulus woodwardi* resolves within a polytomy with *Liomesaspis birtwelli* (Woodward, 1872) and *Alanops magnificus* Racheboeuf, Vannier & Anderson, 2002. *Stilpnocephalus pontebbanus* resolves in a polytomy containing *L. laevis* Raymond, 1944 and *L. leonardensis* Tasch, 1961 and the branch leading to *L. birtwelli*, *A*. *magnificus* and *P. woodwardi*. The autapomorphies that characterize this clade are the reduction or absence of the genal spine, a round thoracetron with limited to no expression of tergal boundaries, and the lack of movable or fixed thoracetronic spines.

### Morphometric results

**Figure 16:**
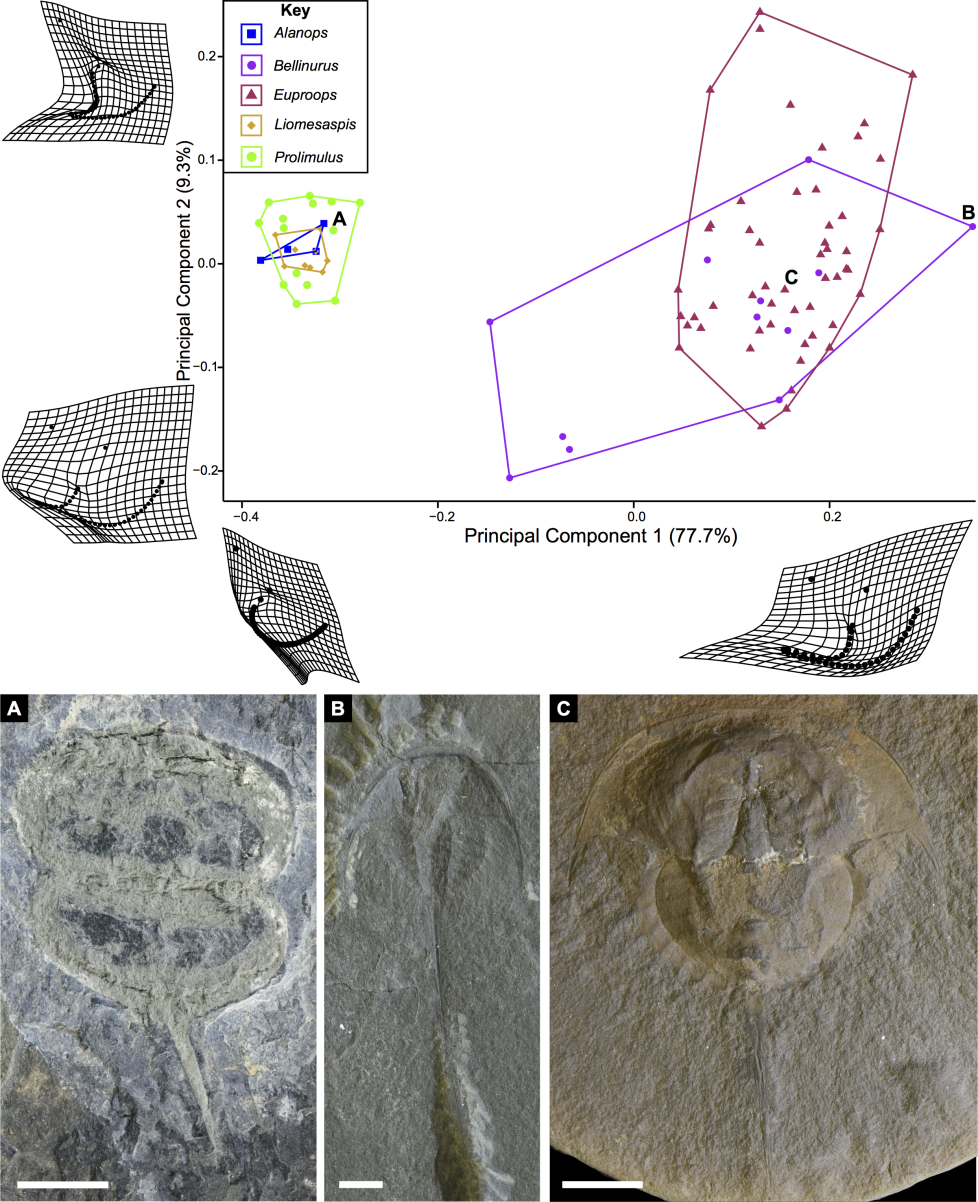
PCA plot of Belinurina morphospace showing PC1 and PC2 following generic assignment presented in Bicknell & Pates (2020). Species with genal spines are located in PC1 space greater than −0.1. *Prolimulus woodwardi* and related species are located in more negative PC1 space. (A) *Prolimulus woodwardi* from the Pennsylvanian (Moscovian)-aged Kladno Formation. NMH L Me 142. (B) *Belinurus* c.f. *truemani* Dix & Pringle, 1929 from the Pennsylvanian (Yeadonian)-aged Sprockhövel Formation, Germany. SMF.Viii.314. (C) *Euproops danae*, from the Pennsylvanian (Moscovian)-aged Mazon Creek *Konservat-Lagerstätte*, Carbondale Formation, USA. YPM IP 50659. Scale bars: 5 mm. Image credit: (A, C): Russell Bicknell. (B): Mónica Solórzano-Kraemer.

**Figure 17:**
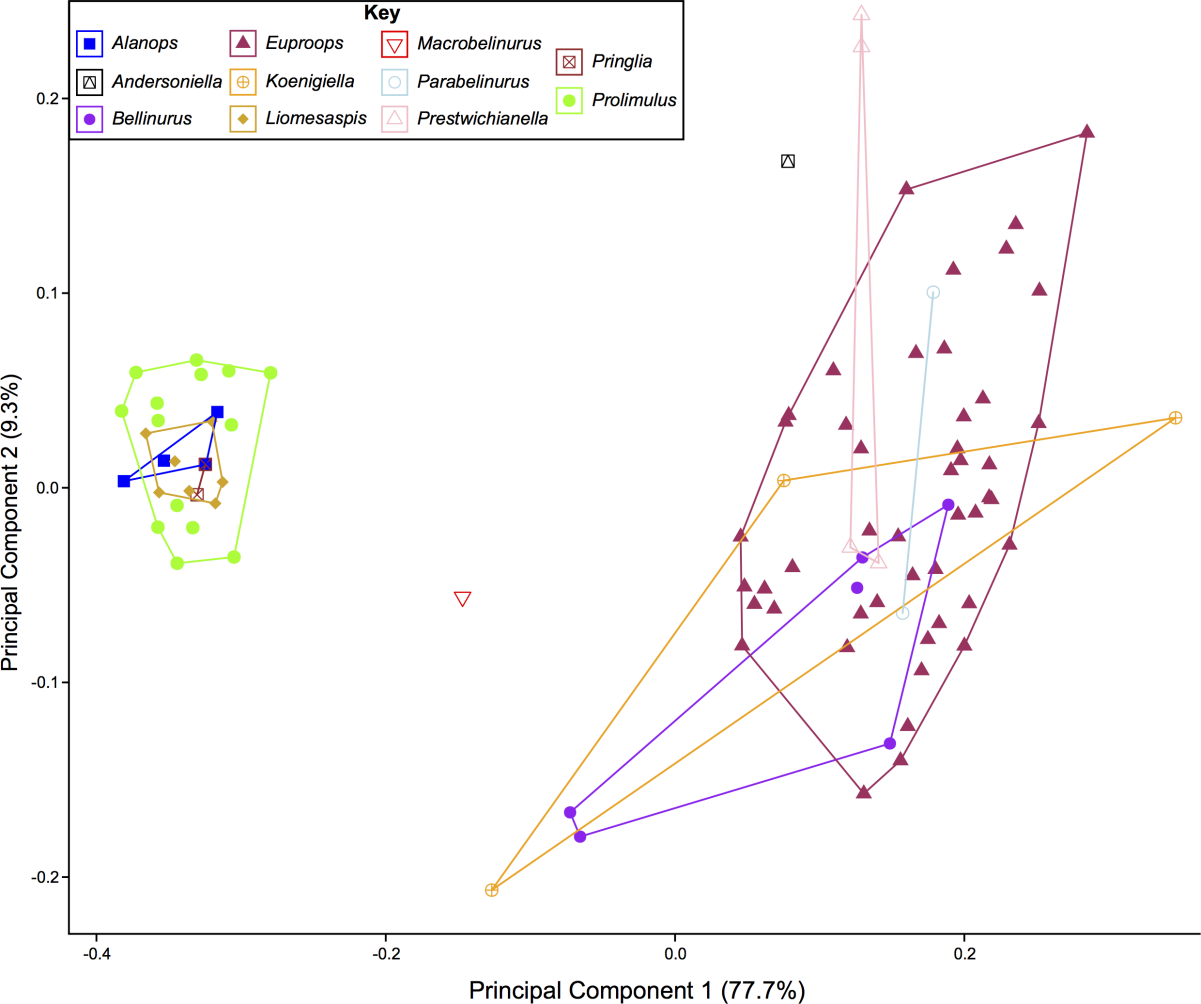
PCA plot of Belinurina morphospace showing PC1 and PC2 following generic assignment presented in Lamsdell (2020b).

The PCA plots illustrate the generic distribution of species within Belinurina in morphospace (Figs. 16 and 17). PC1 (77.7% shape variation) describes the presence or absence of the genal spine. Species within *Belinurus* and *Euproops* (sensu Bicknell & Pates, 2020) therefore fall into mostly positive PC1 space (Figs. 16B, 16C). By contrast, genera without genal spines—*Alanops*, *Liomesaspis*, and *Prolimulus*—dominate negative PC1 space (Fig. 16A). PC2 (9.3% shape variation) describes the posterior elongation of genal spines. This varies within *Euproops* and *Belinurus*. The specimens without genal spines are located in PC2 space of ∼0, reflecting the lack of that feature. Comparing Figs. 16 and 17, the key differences are the distribution of *Belinurus*, *Koenigiella* Lamsdell, 2020b and *Prestwichianella* Lamsdell, 2020b in morphospace. *Belinurus* has a constrained distribution, while *Koenigiella* and *Prestwichianella* have extensive distributions across PC1 and PC2 respectively. Notably, *Euproops danae*—the only representative of *Euproops sensu* Lamsdell (2020b)—has the largest spread in morphospace.

## Discussion

### Evolutionary framework of *Prolimulus* and kin

Belinurids represent the most successful Carboniferous and Permian xiphosurid group that explored freshwater niches (Lamsdell, 2016; Shpinev & Vasilenko, 2018; Shpinev, 2018; Bicknell, Pates & Botton, 2019). The group also has an exceptional diversity and disparity, which is unusual when compared to the Late Mesozoic and Cenozoic forms (Bicknell, 2019; Bicknell et al., 2019a; Bicknell et al., in press-a). Several attempts to colonize freshwater environments likely drove the Belinurina to evolve features that contrast the ‘typical’ xiphosurid morphology, and added to their extreme diversity and disparity (Lamsdell, 2016; Lamsdell, 2020a). Furthermore, freshwater environments can only sustain small populations (Wang et al., 2019) compared to marine conditions and are susceptible to isolating small populations (see DeWoody & Avise, 2000). Allopatric speciation clearly played a central role in the belinurid radiation (Lamsdell, 2016; Lamsdell, 2020a) and permitted innovative characters to be fixed within newly established populations.

The importance of heterochrony during xiphosurid evolution has recently been considered by coding such characters in a phylogenetic framework (Lamsdell, 2020a). This work demonstrated (among other points) that belinurid evolution generally reflects paedomorphosis. While Lamsdell (2020a) did not assess *Prolimulus*, paedomorphic evolution no doubt drove the development of species that are located in the same tree space in Fig. 15. The reduced body size, short or vestigial genal spines, rounded thoracetron, and absence of pre-telson epimera (terminal thoracetronic spines) are considered paedomorphic characters in Belinurina (Lamsdell, 2020a). The reduction or absence of genal spines and a prosoma:thoracetron ratio of ∼1:1 observed in *Prolimulus*, *Alanops*, and *Liomesaspis* are also comparable to *Limulus polyphemus* (Linnaeus, 1758) trilobite stages (Fritsch, 1899; Prantl & Pribyl, 1955; Haug & Rötzer, 2018a). However, the fossil genera display fully developed telson spines, unknown to early postembryonic *L. polyphemus* stages (Haug & Rötzer, 2018a). As such, the lack of genal spines and prosoma:thoracetron ratio are likely phylogenetically significant anatomical similarities, and not aspects of ontogeny. This hypothesis is supported by the Racheboeuf, Vannier & Anderson (2002) dataset that illustrated that the main ontogenetic modification in *A. magnificus* is increased size. The presence of juvenile characters in adult individuals of *Prolimulus* and its kin therefore represent a heterochronic event (Gould, 1977; Klingenberg, 1998). Given this unique combination of characteristics, one might consider erecting a clade to house these notably paedomorphic species. Indeed, Raymond (1944) had erected Liomesaspidae to contain these forms; however, this group is not used anymore. Furthermore, given the convoluted relationships between members of Belinurina, it seems unwise to re-introduce terminology. When phylogenetic and taxonomic relationships within the Belinurina are organized, it may then be pertinent to re-erect a higher order group.

Lamsdell (2020b, p. 17) suggested that *Prolimulus* “strong[s] affinity to *Alanops* and *Pringlia*, and there could be an argument for synonymizing *Prolimulus* with one of these genera”. We disagree with this suggestion based on our observations here. The morphology of the *Prolimulus* thoracetron-telson articulation differs from *Alanops* and *Pringlia* (here considered synonymous with *Liomesaspis*, following the more conservative Anderson & Selden, 1997, and amount of overlap in morphospace; Figs. 13, 17). Furthermore, a ‘thoracetronic boss’ is not observed in *Prolimulus* and the telson likely inserted in the thoracetronic doublure, through a telson notch (Figs. 6, 12 and 14). Regardless, if any synonymy were valid, *Alanops* or *Liomesaspis* would be synonymized with *Prolimulus* (not *vice versa*) as the Czech material has taxonomic priority.

### Ethology

Clusters of extinct arthropods in the fossil record were considered evidence of biological activities (such as gregarious behavior) or traces of digestive processes (bromalite), as opposed to taphonomic artifacts (Speyer & Brett, 1985; Karim & Westrop, 2002; Paterson et al., 2008; Brett et al., 2012; Brett, 2015; Bicknell, Pates & Botton, 2019). Specimens on the sample NM Me 108 (Fig. 7) may represent such gregarious behavior. The lacustrine nature of the sapropelic coal suggest minimal physical disturbance; the individuals were therefore likely not accumulated by currents or other physical factors. As the assemblage is monospecific and has a uniform size distribution, defensive behavior can also be excluded. Interpreting this assemblage as a bromalite is also less parsimonious as there is no evidence of digestion, nor does the cluster conform to the morphology of regurgitalites, coprolites, or cololites (Hunt, 1992). We therefore suggest that either a moulting or mating event best explains the cluster. Horseshoe crab clustering is well documented in extant species (Shuster Jr, 1982; Brockmann, 1990; Brockmann, 2003; Brockmann, Nguyen & Potts, 2000; Brockmann et al., 2015); however, exceptionally rare in the horseshoe crab fossil record. Indeed, the only evidence is one possible *Euproops danae* (Meek & Worthen, 1865) cluster (Ambrose & Romano, 1972; Fisher, 1979; Bicknell, Pates & Botton, 2019). NM Me 108 (Fig. 7) therefore illustrates that clustering was potentially more common than previously thought and was employed by multiple belinurid genera.

### Epibiotic organism associated with Prolimulus

Adult extant xiphosurids often experience interactions with epibionts (Patil & Anil, 2000; Shuster Jr, Botton & Keinath, 2003), while immature individuals often lack evidence of epibiotic fauna (Allee, 1923; Shuster Jr, 1957). This difference reflects frequent moulting by younger individuals, an event that removes any communities attached to the exoskeleton (Shuster Jr & Sekiguchi, 2003). Conversely, moulting events decrease drastically when the animals reach the sexual maturity, such that adult horseshoe crabs may have as few as one moult per year (Carmichael, Rutecki & Valiela, 2003). This infrequency of moulting events allows ectocommensal organisms to colonize the dorsal exoskeleton of adult horseshoe crabs. The presence and distribution of epibionts in the fossil record could therefore be used to infer developmental stages in fossil xiphosurids. Possible parasitic interaction between *Prolimulus woodwardi* (host) and *Spiroglyphus vorax* (parasite, serpulid annelid, or microconchid Taylor & Vinn, 2006) has been suggested (Prantl & Pribyl, 1955). The abundance of *S. vorax* on the studied specimens (Figs. 4A, 4B, 4D; 6A, 6B) suggests that *P. woodwardi* individuals had reached the sexual maturity and the examined population therefore represented fully adult individuals. Such evidence adds to the growing record of potential epibiotic and parasitic relationships preserved within the fossil record (see Conway Morris, 1981; Huntley & DeBaets, 2015; Klompmaker & Boxshall, 2015; Leung, 2017; Zhang et al., 2020).

### Comparing morphology and phylogeny of Belinurina

Xiphosurid morphospace is dominated by extreme shapes; often hypertrophied genal spines. (Bicknell, 2019; Bicknell et al., 2019b; Bicknell & Pates, 2019). Here, we demonstrate this condition by examining exclusively belinurid species: the constructed morphospace is polarized by species with genal spines (e.g., *Euproops* and *Belinurus*) and those lacking the morphology (e.g., *Prolimulus*, *Alanops*). Although morphospace is impacted by taphonomic modification of fossils (Kammerer et al., 2020), this is not apparent within the first two PCs (Bicknell et al., 2019b). Furthermore, as the cuticular xiphosurid exoskeleton requires exceptional preservation conditions, these fossils are seldom subject to the tectonic strain observed in trilobites (Cooper, 1990; Hughes & Jell, 1992).

Comparing the distribution of genera Bicknell & Pates (2020) with Lamsdell (2020b) allows the taxonomic framework based on phylogenetic topology to be examined and scrutinized. The position of *Macrobelinurus* Lamsdell, 2020b and *Andersoniella* Lamsdell, 2020b specimens in morphospace separate from the other clusters strongly supports the validity of these genera. Conversely, the overlap of *Parabelinuris* Lamsdell, 2020b with *Belinurus* and *Euproops* suggests that *Parabelinuris* represents over-splitting of the traditional genera (sensu Bicknell & Pates, 2020). Finally, the large spread of *Koenigiella* and *Prestwichianella* across *Belinurus* and *Euproops* suggests that either these new genera have large morphological variation, or are congeneric with *Belinurus* and *Euproops*. This over-splitting may represent the unfortunate compartmentalization of ontogenetic stages as *Belinurus* and *Euproops* taxa are may record the same ontogenetic trajectory (Haug & Haug, 2020). Regardless, more specimens of all genera are required for this morphospace to be more completely understood and to test the phylogenetic hypotheses of Lamsdell (2020b). Furthermore, a thorough taxonomic revision of the group is needed; a work that should illustrate the range of genera, comparable to Bicknell et al. (in press). Finally, and most importantly, a novel phylogenetic matrix should be constructed in tandem with such a treatise to document independently the convoluted taxonomic record of Belinurina.

## Conclusion

Revision of *Prolimulus woodwardi*, coupled with phylogenetic and geometric morphometric analyses of belinurids, highlighted a diverse clade within Belinurina*.* These species without genal spines all share highly accentuated paedomorphic characters, such as vestigial genal spines, and are representative of paedomorphic evolution. A slab of multiple *P. woodwardi* individuals demonstrates new evidence for Carboniferous horseshoe crab ecology, revealing possible gregarious behavior, and further data on the deep origin xiphosurid clustering. Taken together, the examination presented here demonstrates the morphological variation and ecological conditions that permitted successful colonization of freshwater environments by Carboniferous horseshoe crabs.

##  Supplemental Information

10.7717/peerj.10980/supp-1 Supplemental Information 1 Phylogenetic matrix Originally presented in Lamsdell (2016).

10.7717/peerj.10980/supp-2 Supplemental Information 2 Specimens digitized for geometric morphometrics

10.7717/peerj.10980/supp-3 Supplemental Information 3 PCA results Includes family and generic assignment of specimens.

## INSTITUTIONAL ABBREVIATIONS

MBA: Museum für Naturkunde, Leibniz-Institut, Berlin, Germany
MCZ: Museum of Comparative Zoology, Harvard University, Cambridge, MA, USA
MNHN: Museum National d’Histoire Naturelle of Paris, Paris, France.
MPT: Museo Archeologico e Naturalistico, Tarcento, Udine, Italy
NHMUK PI: Natural History Museum, London, UK
NM: National Museum, Paleozoic Invertebrate collection, Prague, Czech Republic
SMF: Forschungsinstitut Senckenberg, Frankfurt am Main, Germany
YPM IP: Division of Invertebrate Paleontology in the Yale Peabody Museum, New Haven, Connecticut, USA

## Additional Information and Declarations

### Data Availability

The following information was supplied regarding data availability: Raw data are available in the Supplemental Files.
